# Theoretical screening of bistriazole-derived energetic salts with high energetic properties and low sensitivity†

**DOI:** 10.1039/c9ra05141d

**Published:** 2019-08-22

**Authors:** Xiao-Hong Li, Cong Zhang, Xue-Hai Ju

**Affiliations:** College of Physics and Engineering, Henan University of Science and Technology Luoyang 471003 China; Key Laboratory of Soft Chemistry and Functional Materials of MOE, School of Chemical Engineering, Nanjing University of Science and Technology Nanjing 210094 P. R. China xhju@njust.edu.cn

## Abstract

We designed four series of energetic anions by replacing nitro group (NO_2_) with trinitromethyl group (C(NO_2_)_3_) or by inserting N-bridging groups (–NH–, –NH–NH–, –N=N–, –N=N(O)–) into the bistriazole frameworks. The properties of 40 energetic salts, based on the bistriazole-derived anions and hydroxylammonium cation, were studied by density functional theory (DFT) and volume-based thermodynamics calculations (VBT). It is found that the newly designed energetic salts have good detonation properties due to their larger nitrogen content and better oxygen balance. And one of their corresponding hydroxylammonium salts exhibits better detonation performance (*D* = 10.06 km s^−1^ and *P* = 48.58 GPa) than CL-20 (*D* = 9.54 km s^−1^ and *P* = 43.36 GPa). Moreover, 10 energetic salts not only exhibit excellent energetic properties superior to CL-20, but also have lower sensitivity than CL-20 (*h*_50_ = 13.81 cm). In addition, we rationally selected salt B6 from the 10 salts to predict its crystal structure under pressures. By converting energetic molecules with excellent detonation properties into energetic ions, some highly bistriazole-derived energetic salts with both excellent performance and low sensitivity could be developed strategically.

## Abstract 2

We designed four series of energetic anions by replacing nitro group (NO_2_) with trinitromethyl group (C(NO_2_)_3_) or by inserting N-bridging groups (–NH–, –NH–NH–, –N=N–, –N=N(O)–) into the bistriazole frameworks.

## Graphical abstract

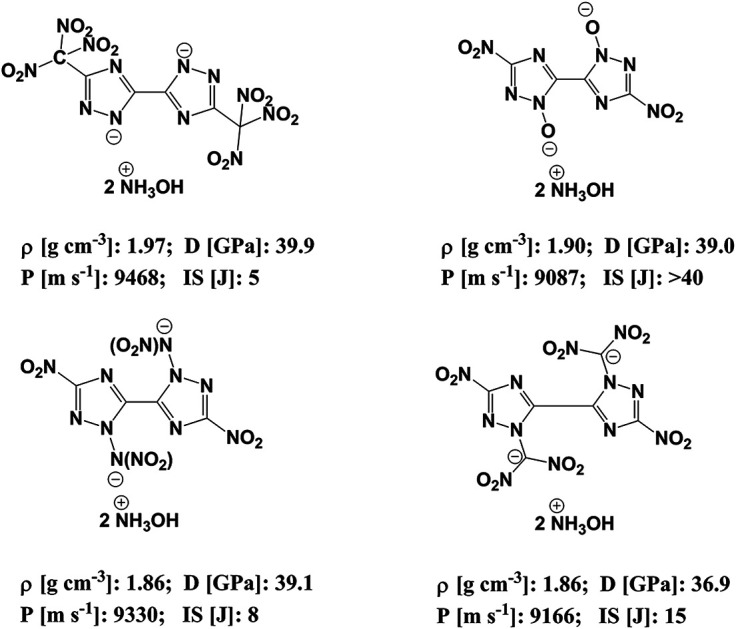

## 1. Introduction

Along with the growing demand for a new generation of advanced energetic materials to replace conventional explosives, extensive studies have been focused on finding high energy density materials (HEDMs) with lower impact sensitivity and better detonation performance as well as being environmentally friendly.^1,2^ Over the past years, energetic ionic salts as a new class of energetic materials have received a substantial amount of interest because they are environmentally friendly and have lower vapor pressures, higher heats of formation, and enhanced thermal stabilities as compared to conventional nonionic analogs.^3,4^ Since the cations and anions in the salts can be modified independently, we can produce a large amount of different ionic salts by combining different potential cations and anions.

In the design of high energy density molecules, combining various energetic substituents with a variety of backbones is the most popular strategy.^5^ In the parent structure of nitrogen-enriched compounds, the nitrogen content of the triazole precursor is relatively high and the triazole structure provides convenience for the substitution with other energetic groups. Among all energetic groups, nitro group, nitramine group and trinitromethyl group are often the preferred groups since they can not only increase the nitrogen content, but also increase the oxygen balance of the energetic molecules. Therefore, polynitro-substituted triazole derivatives have attracted wide attention of researchers due to their excellent detonation properties.^6–8^ However, the impact sensitivity of such triazole derivatives is often limited by the introduction of excessive nitro groups.

**Fig. 1:**
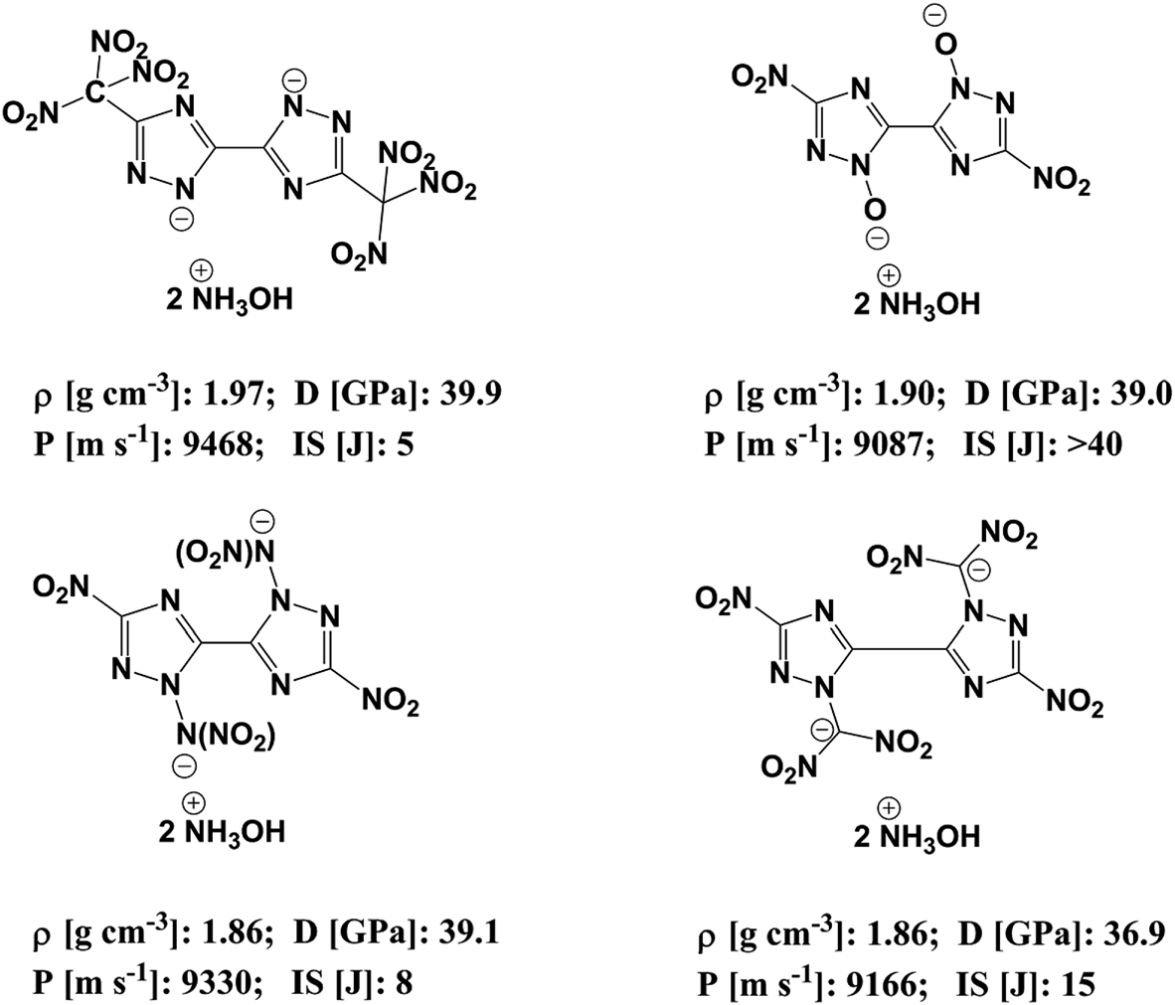
Salts containing bistriazole-derived anions and hydroxylammonium (*D*: detonation pressure; *P*: detonation velocity from; IS: impact sensitivity. Data are from literature^9–12^).

In order to reduce the impact sensitivity of energetic molecules, we can convert them into ions. Therefore, if we are able to convert energetic molecules with excellent detonation properties into energetic ions, we can endow energetic ionic salts with good detonation performance and impact sensitivity simultaneously. At present, many studies of energetic salts are concentrated on the structures and properties of nitrogen-enriched anions. To the best of our knowledge, the deprotonation of triazole is mainly in four positions: one is the *N*-position on the triazole (–[N^⊖^]–); the second is the hydroxyl group of the triazole (–[N(O^⊖^)]–); the third is the nitroamine group on the triazole (–[N(N^⊖^NO_2_)]–); the fourth is the dinitromethyl group on the triazole (–[N(C^⊖^(NO_2_)_2_)]–). Fig. 1 exhibits the salts of bistriazole anions displaying these positions.^9–13^ We can find that all these bistriazole-derived salts have good detonation performance and low impact sensitivity. However, the role of these deprotonated groups in the bistriazole-derived salts has not been studied systematically.

What's more, the addition of nitrogen-containing bridging groups can increase the heat of formation, thereby improves the detonation performance.^14,15^ Therefore, connecting two triazole molecules with these bridging groups will help to construct energetic molecules with excellent detonation properties.

**Fig. 2:**
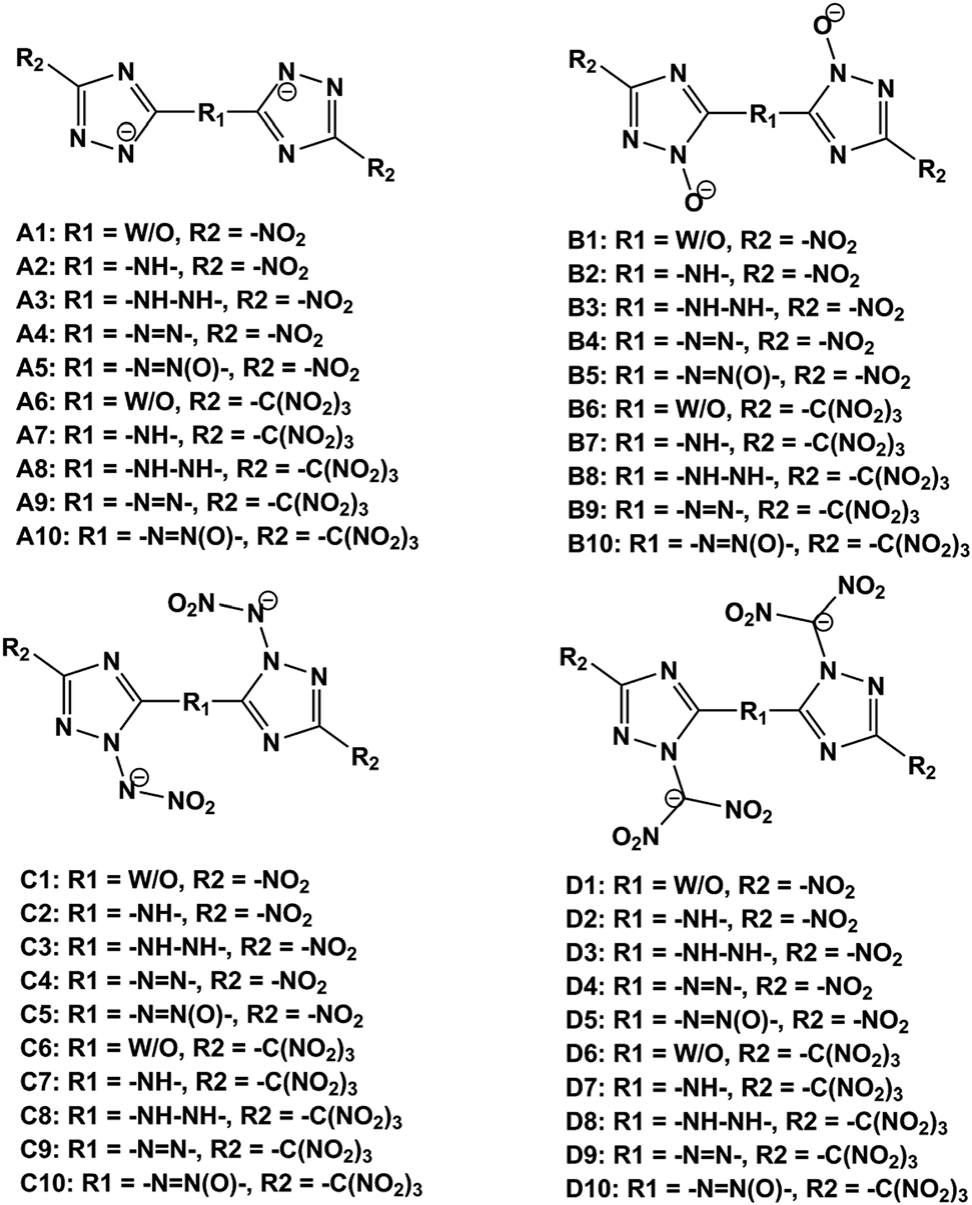
The frameworks of bistriazole-derived anions.

In this work, we designed a series of hydroxylammonium bistriazole ionic salts (Fig. 2) and performed density functional theory (DFT)^16^ and volume-based thermodynamics (VBT)^17^ calculations to obtain their densities, HOFs, energetic properties, impact sensitivities and Gibbs free energies of formation. Our work will help to elucidate the role of different deprotonated groups (–[N^⊖^]–, –[N(O^⊖^)]–, –[N(N^⊖^NO_2_)]–, –[N(C^⊖^ (NO_2_)_2_)]–), nitro/trinitro methyl groups (–NO_2_, –C(NO_2_)_3_) and N-bridging groups (–NH–, –NH–NH–, –N=N–, –N=N(O)–) in the design of hydroxylammonium bistriazole salts. In addition, we hope to validate our design strategy that converting energetic molecules with excellent explosive properties into energetic ions for the search of high-energy and insensitive materials. What's more, in order to promote the experimental study, we selected one energetic salt after comprehensively comparing the detonation performance, impact sensitivity and easiness of synthesis of all energetic salts, and then predicted its crystal structures under different high pressures.

## 2. Computational method

All quantum chemical calculations were performed with the Gaussian 09 program package (G09).^18^ The geometries of all bistriazole-based salts were fully optimized at the DFT(B3LYP)/6-31++G(d) level which was successfully applied to calculate the properties of energetic ionic salts. All of the optimized structures were characterized to be true local energy minima on the potential energy surface with no imaginary frequencies.

### 2.1 Calculations of density

For energetic materials, the density can directly affect its detonation performance. Therefore, some approaches have been evolved in order to predict the crystal density more accurately.^19–21^ According to these methods, we need to take intermolecular interactions within the crystal into account since the traditional *M*/*V* procedure often leads to large errors without considering that. Thus, the crystal density of ionic salts can be calculated by the following formula:^22^

1 *ρ* (g cm^−3^) = *α*(*M*/*V*) + *β*(*V*_S_^+^/*A*_S_^+^) + *γ*(*V*_S_^−^/*A*_S_^−^) + *δ*

where, *M* is the chemical formula mass of the ionic compound and *V* is the total volume of that substance. *A*_S_^+^ is the electrostatic potential of the cation, *V*_S_^+^ is the average potential of the cation, and *A*_S_^−^ and *V*_S_^−^ are those of anions. The coefficients *α*, *β*, *γ*, and *δ* in eqn (1) are taken from the ref. 21. Through the Multiwfn program,^23^ we could get these surface electrostatic properties.

For the ionic crystal with formula unit M_*p*_X_*q*_, its volume is simply the sum of the volumes of the ions contained in the formula unit:

2 *V* = *pV*_M_^+^ + *qV*_X_^−^

where, the subscripts M and X denote the cation and anion, respectively. Due to the volumes of individual ions can be obtained by using DFT procedure, we used eqn (2) to calculate the volumes of the salts.

### 2.2 Calculations of heats of formation

Through Born–Haber cycle (Scheme S1†), we predicted the heats of formation of energetic salts and the calculation procedure can be simplified by eqn (3):3
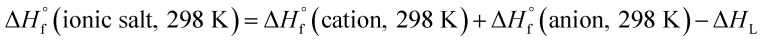
in above equation, Δ*H*_L_ is the lattice energy of the salts which can be predicted by the formula proposed by Jenkins^24^*et al.* as:

4 Δ*H*_L_ = *U*_POT_ + [*p*(*n*_M_/2 − 2) + *q*(*n*_X_/2 − 2)]*RT*

where, the *n*_M_ and *n*_X_ values are equal to three for our predicted ions. *U*_POT_ (kJ mol^−1^) is the lattice potential energy which can be calculated by the following expression:

5 *U*_POT_ = *γ*(*ρ*/*M*)^1/3^ + *δ*

where *ρ* (g cm^−3^) is the predicted density, *M* is the chemical formula mass (g mol^−1^) of salts and the coefficients *γ* and *δ* for our designed 2 : 1 salts are 8735.6 kJ mol^−1^ cm and −178.8 kJ mol^−1^, respectively.^25^

Meanwhile, isodesmic reactions (Scheme S2†) of these cage anions and ammonium-based cations were used to predict their HOFs at 298 K. For the ions and some small molecules whose experimental HOFs are not available, their HOFs were calculated by using protonation and atomization reactions, respectively (with G4 theory).^26^

### 2.3 Calculations of detonation properties

The performance parameters of energetic materials, detonation velocity (*D* in km s^−1^) and detonation pressure (*P* in GPa), were computed using the empirical Kamlet–Jacobs as shown in the following equations:^27^

6 *D* = 1.01(*NM*^1/2^*Q*^1/2^)^1/2^(1 + 1.30*ρ*)

7 *P* = 1.558*ρ*^2^*NM*^1/2^*Q*^1/2^

in which, *N* is moles of explosive gas, *M* is the average molecular weight of these gases (g mol^−1^), *Q* (cal g^−1^) is related to the HOF difference between the product and the reactant calculated from the exothermic principle, and *ρ* stands for the predicted density of salts (g cm^−3^).

### 2.4 Calculations of impact sensitivity

As the impact sensitivity become increasingly important for energetic materials, there is a simple method developed by Keshavarz^28^ to estimate the *h*_50_ (cm) as follows:

8 (log *h*_50_)_core_ = −0.584 + 61.62*a* + 21.53*b* + 27.96*c*

9 (log *h*_50_) = (log *h*_50_)_core_ + 84.47*F*^+^/MW − 147.1*F*^−^/MW

where, (log *h*_50_)_core_ is calculated based on the elemental composition: *a*, *b*, *c* represent the composition of carbon, hydrogen and nitrogen, respectively. MW is the chemical formula mass (g mol^−1^) of the compound. The data of *F*^+^ and *F*^−^ were selected from literature.^27^

### 2.5 Calculations of Gibbs free energies of reactions

The Born–Haber thermodynamic cycle for the formation reactions of the salts (Scheme S3†) was designed to predict the Δ*G*_rxn_. And the equation is as follows:

10 Δ*G*_rxn_ (salt) = Δ*H*_rxn_ (salt) − *T*Δ*S*_rxn_ (salt)

The total enthalpy of the generalized reactions for the titled salts was predicted by eqn (11):

11 Δ*H*_rxn_ (salt) = Δ*H*_1_ + Δ*H*_2_ + Δ*H*_3_

where Δ*H*_1_ is the enthalpy change of the deprotonation process of the anion's prototype molecules; Δ*H*_2_ is the enthalpy change in protonation of RNH_2_; Δ*H*_3_ is directly related to the lattice energy when the cation and anion combine into the salts.

Then, for the entropy change of this process was predicted by the following equation:

12 Δ*S*_rxn_ (salt) = *S*_salt_ − *S*_proton-donor_ − *S*_proton-acceptor_

where *S*_proton-donor_ and *S*_proton-acceptor_ are the entropies for the prototype molecules of anion and cation, respectively. *S*_salt_ is the evaluated entropy which was predicted by using the formula developed by Glasser and Jenkins^29^ for organic solids:

13 *S*_salt_ (298 K) = 1.285(*M*/*ρ*) + 57

### 2.6 Prediction of crystal structures

When the structures of the ionic compounds were optimized by G09, their electrostatic potentials (ESP), charges were obtained by DMol3 module in Materials Studio (MS)^30^ and embedded into each atom. Then, the Polymorph Predictor (PP) module of MS was applied to predict crystal structures with Dreiding force field, which is considered to be more appropriate force fields for crystal prediction.^31^ According to the Cambridge Structural Database, about 90% of all organic and organometallic crystal structures are covered by the 17 most frequent space groups. For the ionic compounds, the PP calculations were restricted to the nine most probable space groups, *P*2_1_/*c*, *Pna*2_1_, *P*2_1_2_1_2_1_, *Pbca*, *P*2_1_, *C*2/*c*, *Cc*, *C*2 and *P*1̄.^32^ The generated structures for all nine space groups were arranged according to their energies in the list starting from the structure with the lowest energy. Finally, the predicted crystal structures with the lowest energies were selected and the properties of the crystal compounds under different pressures (0 GPa to 40 GPa) were predicted by the Cambridge Sequential Total Energy Package (CASTEP) module in MS using GGA-PBE functional.

## 3. Result and discussion

### 3.1 Crystal density

Density is one of the most significant factor for energetic materials as higher density means that more energy will be packed per unit volume in these materials.^33^ Specifically, crystal density can directly influence the detonation performance which is shown in the Kamlet–Jacobs equation. By replacing the nitro group or inserting the different bridges, the structure of bistriazole was changed, which will cause different effect in density. In addition, there is also an effect on the density of title salts when the bistriazole-derived frame contains different deprotonated groups. The title salts possess densities ranging from 1.71 to 2.07 g cm^−3^ (Table S1†). By comparing our calculated values with the experimental values of the available energetic salts, we can find that the largest difference is 0.13 g cm^−3^, and the smallest difference is only 0.04 g cm^−3^. Therefore, our calculated values are reliable in this work.

**Fig. 3:**
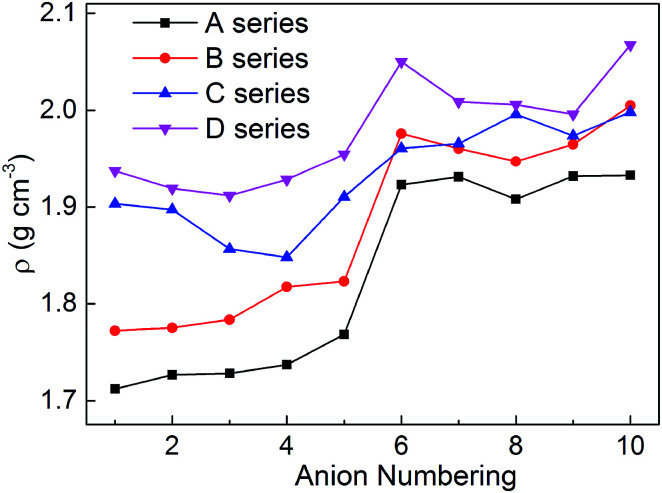
Comparison of the densities of the salts.

As shown in Fig. 3, when comparing the change trend of series A with other series, the variation of density for series B is a bit inconsistent, while series C and D are completely different. What's more, the densities for each series are in the following order: D > C > B > A. This indicates that the bistriazole moiety containing –C^⊖^(NO_2_)_2_ is the most efficient framework for enhancing the densities of the title salts. In addition, for the same series, the densities of salts with anion numbering 6–10 are generally larger than salts with anion numbering 1–5, which illustrates that –C(NO_2_)_3_ can greatly improve the densities of salts. And by comparing the densities of salts with anion numbering 6–10 of the same series, the density of salts with anion numbering 10 often has a larger density, which means that the linkage –N=N(O)– can increase the densities of title salts. Therefore, the bistriazole frame containing deprotonated dinitromethyl group (–C^⊖^(NO_2_)_2_), –C(NO_2_)_3_ group and –N=N(O)– linkage is the best anion for increasing the density of hydroxylammonium bistriazole-derived salts.

### 3.2 Heats of formation (HOFs)

The heats of formation usually represents the energy content of the energetic compounds, which are derived from their molecular skeletons and substituents.^34^ Based on the related atomization reactions and isodesmic reactions, we calculated HOF values (Table S2†) of small molecules and ions, and then the heats of formation of title salts (Table S3†). And all title salts exhibit high positive HOFs (157.10–869.15 kJ mol^−1^). Therein, we compared our calculated HOF values with the experimental HOF values of the existed energetic salts, and we can find that the two values are similar. This means that our calculated methods are reliable in this work. To verify the reliability of the method, we further calculated the HOF of B6 at the B3LYP/aug-cc-pVTZ level. The HOF values are 380.13 kJ mol^−1^ and 358.61 kJ mol^−1^ at the B3LYP/6-31++G(d) and B3LYP/aug-cc-pVTZ levels, respectively. The relative deviation of 6% indicates that the 6-31++G(d) basis set is appropriate for balancing the computational accuracy and efficiency.

**Fig. 4:**
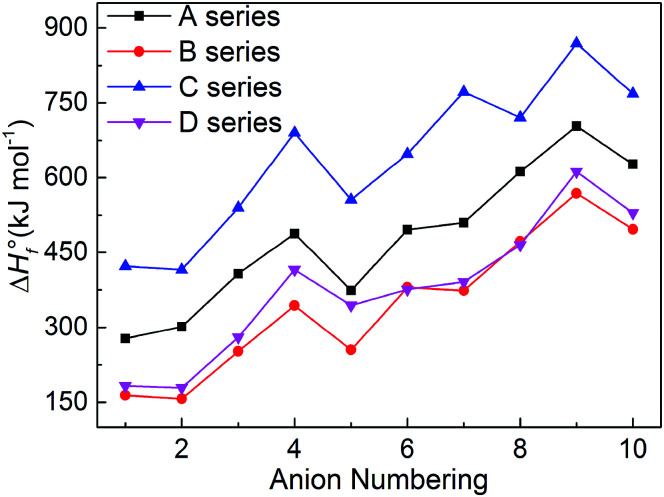
Comparison of the HOFs of the salts.

It is seen in Fig. 4 that the variation trends of the salts of series A and B are unanimous which shows that the bistriazole frame containing small deprotonated groups (like –NO^⊖^) hardly affect the change trends of the HOFs of title salts. When comparing with series A, the HOFs of series C is generally larger than those of A, while for series B and D, the situation is quite different. Therefore, it is good to choose the bistriazole frame containing deprotonated dinitromethyl group (–C^⊖^(NO_2_)_2_) as an anion for energetic salts in order to obtain excellent HOFs. When the other two groups are the same, the –C(NO_2_)_3_ often has better effect than the –NO_2_ for enhancing the HOFs of title salts. In addition, HOF values of salts with the fourth anion numbering is always larger than the salts with anion numbering 1–5, and the same rule appears for salts with anion numbering 6–10 (the fourth order changes into 9^th^ here), which indicates that the –N=N– is the most potential linkage for increasing the HOFs among five N-bridging groups. In a word, choosing the bistriazole frame containing deprotonated dinitromethyl group (–C^⊖^(NO_2_)_2_), replacing nitro group (NO_2_) with –C(NO_2_)_3_ group and inserting –N=N(O)– linkage into the framework is helpful for improving the HOFs of its salts.

### 3.3 Energetic properties

The detonation velocity and detonation pressure are the most important parameters to access energetic materials. Since Kamlet–Jacobs equations have been proved to reliable and widely used to compute the detonation properties, we calculated the heats of detonation (*Q*), detonation velocities (*D*) and pressures (*P*) according to our predicted densities and HOFs. And the values of detonation parameters, nitrogen content (NC), oxygen balance (OB) of hydroxylammonium bistriazole-derived salts were summarized in Table 1. Meanwhile, the detonation parameters of CL-20 were also selected for comparing. The calculated *Q*, *D* and *P* of CL-20 are consistent with the experimental values and the calculated *D* and *P* of the existed energetic salts are also similar to its experimental values. This indicates that our calculated results for the title salts are very reliable in this work.

**Table 1:** Predicted heats of detonation (*Q*), detonation velocities (*D*) and pressures (*P*), nitrogen content (NC), oxygen balance (OB) and impact sensitivity (*h*_50_) for the salts

Salts	NC (%)	OB^a^ (%)	*Q* (cal g^−1^)	*D* (km s^−1^)	*P* (GPa)	*h*_50_ (cm)
A1	48	−32.87	1340.88	8.21	28.98	64.12
A2	50	−33.87	1311.30	8.25	29.40	71.02
A3	52	−34.78	1345.72	8.34	30.08	77.92
A4	52	−29.99	1380.86	8.38	30.51	59.23
A5	50	−23.81	1373.69	8.52	31.88	45.75
A6	39	−6.40	1639.35	9.45 (9.47)^b^	41.21 (39.9)^b^	19.17
A7	41	−7.77	1608.67	9.47	41.41	21.10
A8	42	−9.06	1619.19	9.42	40.75	23.10
A9	42	−6.06	1646.28	9.51	41.84	19.48
A10	41	−2.94	1650.80	9.55	42.17	17.16
B1	43	−19.75	1414.79	8.62 (9.0)^c^	32.63 (39.0)^c^	37.23
B2	45	−21.24	1363.22	8.58	32.38	41.83
B3	47	−22.60	1384.60	8.67	33.15	46.55
B4	48	−18.18	1424.21	8.81	34.61	36.17
B5	46	−13.04	1432.23	8.88	35.22	29.19
B6	37	0.00	1665.43 (1655.77)^d^	9.74 (9.78)^d^	44.38 (45.05)^d^	14.80
B7	38	−1.46	1626.83	9.65	43.39	16.32
B8	40	−2.85	1634.91	9.63	43.10	17.90
B9	40	0.00	1662.59	9.71	43.97	15.22
B10	39	2.78	1668.32	9.88	46.08	13.60
C1	48	−7.77	1490.50	9.29 (9.33)^e^	39.58 (39.1)^e^	24.11
C2	49	−9.37	1446.92	9.23	38.94	26.85
C3	51	−10.86	1477.31	9.15	37.85	29.69
C4	51	−7.27	1541.18	9.19	38.03	24.22
C5	49	−3.51	1519.59	9.40	40.60	20.66
C6	41	5.16	1683.85	9.78	44.57	12.64
C7	42	3.78	1699.19	9.84	45.17	13.80
C8	43	2.46	1649.36	9.89	46.05	14.00
C9	43	4.94	1692.64	9.85	45.37	13.04
C10	42	7.23	1686.33	9.94	46.59	11.86
D1	39	−6.40	1489.92	9.28 (9.17)^f^	39.88 (36.9)^f^	19.17
D2	41	−7.77	1455.09	9.19	38.89	21.10
D3	42	−15.09	1375.19	9.00	37.25	23.10
D4	42	−6.06	1515.87	9.31	40.00	19.48
D5	41	−2.94	1526.52	9.44	41.48	17.16
D6	36	4.52	1648.36	9.94	47.26	11.65
D7	37	3.32	1626.56	9.79	45.26	12.60
D8	38	2.17	1624.81	9.79	45.27	13.59
D9	38	4.35	1662.27	9.79	45.09	12.01
D10	37	6.38	1663.22	10.06	48.58	11.07
HMX	38	−21.62	1597.4	8.9 (8.8)^g^	34.8 (34.7)^g^	29.17 (26–33)^g^
RDX	38	−21.62	1633.9	9.3 (9.1)^g^	39.2 (39.0)^g^	31.28 (29–36)^g^
CL-20	38	−10.96	1574.32	9.54 (9.40)^h^	43.36 (42.0)^h^	13.81 (11.94)^h^

a Oxygen balance (%) for C_*a*_H_*b*_O_*c*_N_*d*_: 1600 × (*c* − 2*a* − *b*/2)/*M*_w_, where *M*_w_ is the molecular weight of the corresponding compounds.

b Experimental values in parentheses were from ref. 9.

c Experimental values in parentheses were from ref. 10.

d Using the heat of formation obtained from the B3LYP/aug-cc-pVTZ.

e Experimental values in parentheses were from ref. 11.

f Experimental values in parentheses were from ref. 12.

g The calculated values in parentheses were from ref. 2 and 27.

h The calculated values in parentheses were from ref. 33.

Nitrogen content and oxygen balance are two primary parameters for screening the energetic compounds due to their simple calculation methods. The detonation performance of energetic materials depends on both. Obviously, the larger the nitrogen content is, the better the detonation performance is. And when the oxygen balance is zero, the explosion reaction releases the maximum heat with the strongest capacity to do work. Therefore, an energetic compound with high nitrogen content and near zero oxygen balance will have better detonation performances. From Table 1, it can be seen that all salts has higher nitrogen content than 35%. In addition, for series A and B, the oxygen balance is near zero only for the salts with trinitromethyl group, but for series C and D, the oxygen balance of almost all salts is near zero. This indicates that these salts will have good detonation performance.

**Fig. 5:**
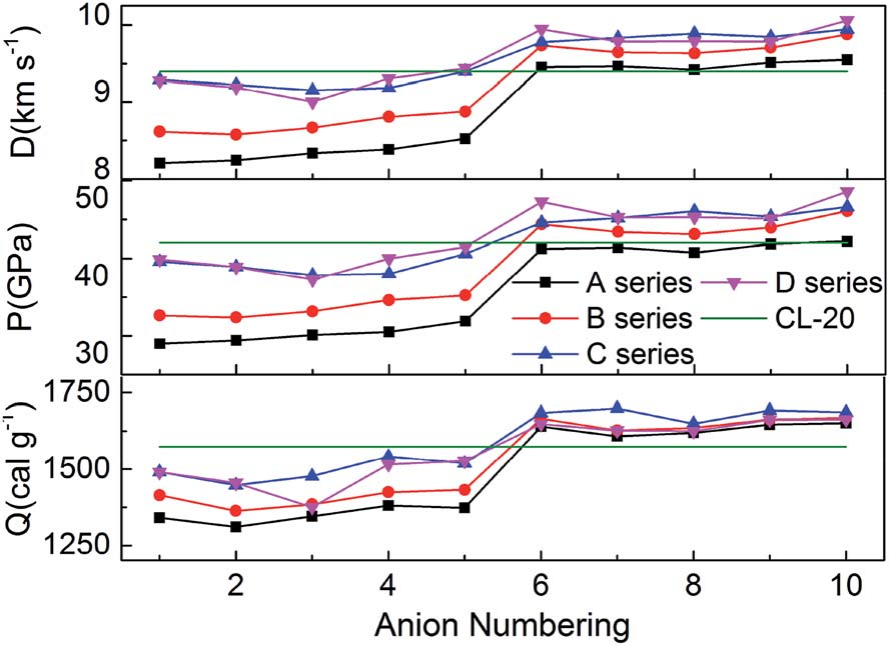
Heats of detonation, detonation velocities, and detonation pressures of the salts.

Fig. 5 represents the comparison of the effects of different anions on *Q*, *D* and *P* of hydroxylammonium bistriazole-derived salts. Because these values depend on the densities and HOFs of title salts, it is easily seen that the variation trends of the salts of series A and series B are unanimous. In addition, the salts of series C and series D usually own the higher *Q*, *D* and *P* than series A and series B. For the four series, the detonation performance of salts with anion numbering 6–10 are generally better than salts with anion numbering 1–5. This means that the introducing of –C(NO_2_)_3_ usually has a great enhancement on the detonation properties of title salts. At the same time, the salts with anion numbering 10 of each series usually have higher detonation properties than the salts with other anion numbering. This is because the –N=N(O)– not only has N=N conjugated structure, but also has N→O oxidation bond. Thus, it has better enhancement for the detonation properties. The *Q*, *D* and *P* of B6 were also calculated from the HOF value and density at the B3LYP/aug-cc-pVTZ level. Variations of *Q*, *D* and *P* between the results from 6-311++G(d) and aug-cc-pVTZ basis sets for B6 are very small.

Overall, almost 19 title salts exhibit better detonation performance than CL-20. The predicted results confirm that the introduction of nitro groups is indeed an effective way to enhance the energetic properties remarkably.

### 3.4 Impact sensitivity

For energetic compounds, impact sensitivity is an important factor to judge their stabilities to external impact.^35^ The impact sensitivity is generally reported as the height in cm, designated *h*_50_. The higher the *h*_50_ is, the less sensitive the explosive is. The calculated *h*_50_ value of CL-20 was also listed in Table 1 for comparison. The evaluated *h*_50_ value of CL-20 is close to the result from literatures, indicating that our predicted results are reliable. Furthermore, the previous literature showed that the experimental values of the impact sensitivity of ionic compounds of TKX-50 and its analog AHTA deviate from the calculated value by only 10.38 and 2.78 cm (with relative deviations of 12.7% and 4.5% from experiments), respectively.^36^ Therefore, Keshavarz's method in eqn (9) is applicable for ionic compounds.

It can be seen from Fig. 7 that most salts has higher *h*_50_ than CL-20, indicating that title salts are insensitive. What's more, series A have the highest *h*_50_ among the four series, the next are series B and series C, and the last one is series D. And the hydroxylammonium NO_2_-substituted bistriazole-derived salts have higher *h*_50_ than corresponding C(NO_2_)_3_-substituted ones. This indicates that the energetic materials are more sensitive when more nitro groups are introduced. Among the bridged bistriazole-derived salts with the same substitutent, the salts with the bridge –NH–NH– possess the highest *h*_50_.

Overall, almost 30 title salts exhibit better impact sensitivity than CL-20. Therein, 10 salts already have better detonation performance than CL-20. The predicted results confirm that converting energetic molecules with excellent detonation properties into energetic ions is a potential way for screening HEDMs with low sensitivity.

### 3.5 Gibbs free energies of formation

**Fig. 6:**
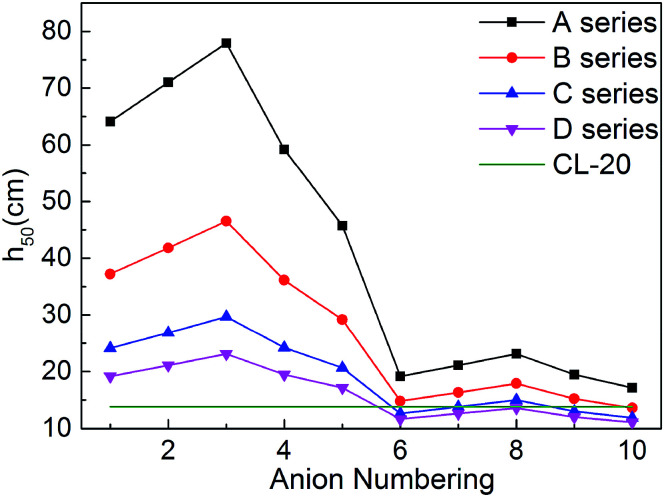
Comparison of the impact sensitivity of the salts.

**Fig. 7:**
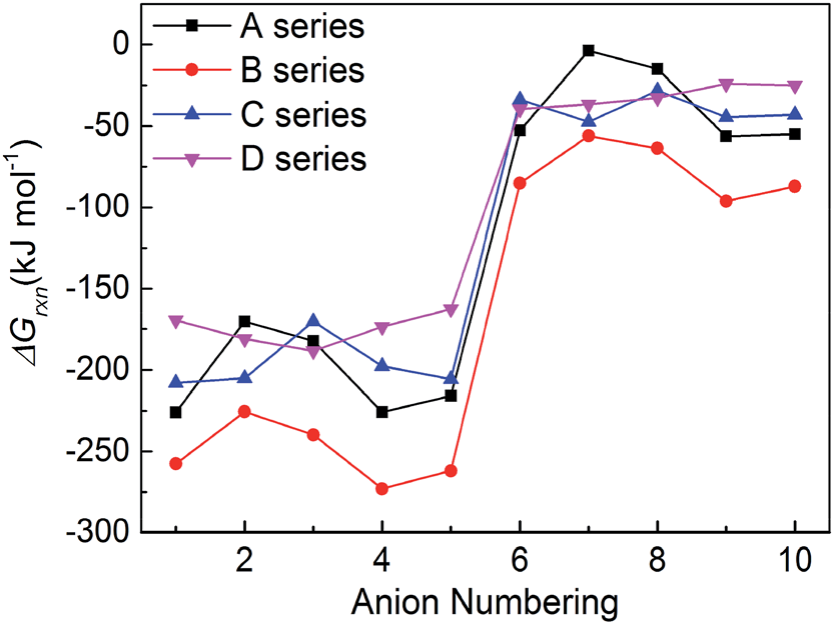
The comparison of the Δ*G*_rxn_ of the salts.

New energetic salts not only should have desirable explosive properties, but also should be easy and cheap to synthesize. The easiness of synthesis can be judged by the Gibbs free energies of formation. The more negative the Δ*G*_rxn_ is, the easier the salt is to form. Fig. 6 shows a comparison of the effects of different anion on the Δ*G*_rxn_ of title salts. It is seen that almost all the title salts have negative Δ*G*_rxn_. This suggests that the salts could be formed spontaneously. Meanwhile, the salts containing the group –C(NO_2_)_3_ always have higher Δ*G*_rxn_ than corresponding NO_2_-substituted ones. This shows that the Δ*G*_rxn_ of the salt can be changed by different frameworks, nitro/trinitro groups and N-bridging groups (Fig. 7).

### 3.6 Prediction of crystal packing

In this section, we predicted the crystal structure of salt B6 since it exhibits better detonation performance, lower sensitivity and easier to synthesize. The force field of Dreiding is used to estimate the crystal structure of B6 since it produces the condensed-phase properties reliable for a lot of organic compounds. It can be seen in Table 2 that the structure with *Cc* symmetry has the lowest energy among all 10 polymorphs. It could be inferred that B6 belongs to the *Cc* space group. Its lattice parameters are predicted to be *a* = 5.49 Å, *b* = 22.43 Å, *c* = 16.37 Å, *α* = 90.0°, *β* = 114.11°, and *γ* = 90.0°. And the optimized crystal structure of the B6 was shown in Fig. S1.†

**Table 2:** Predicted crystal properties of B6^a^

No.	SG^b^	*E*_total_ c	*a*	*b*	*c*	*α*	*β*	*γ*
1	*Cc*	−241.95	5.49	22.43	16.37	90.00	114.11	90.00
2	*Pna*2_1_	−240.51	7.23	15.76	16.22	90.00	90.00	90.00
3	*P*2_1_	−240.30	12.97	15.13	5.75	90.00	123.18	90.00
4	*P*2_1_/*c*	−239.74	28.55	9.38	11.58	90.00	143.69	90.00
5	*P*1̄	−239.65	9.94	20.06	5.53	85.78	71.16	63.91
6	*P*1̄	−239.45	11.18	12.37	8.35	80.37	108.23	70.03
7	*Cc*	−239.45	5.53	21.64	16.99	90.00	110.29	90.00
8	*P*2_1_2_1_2_1_	−239.42	18.71	7.84	12.36	90.00	90.00	90.00
9	*P*2_1_	−239.35	11.95	15.43	5.74	90.00	60.84	90.00
10	*Cc*	−238.93	5.51	20.81	17.56	90.00	108.89	90.00

a Cell parameters are in Å or degree.

b SG = space group.

c Total energy per molecule in kcal mol^−1^.

### 3.7 Prediction of crystal structures under different pressures

**Fig. 8:**
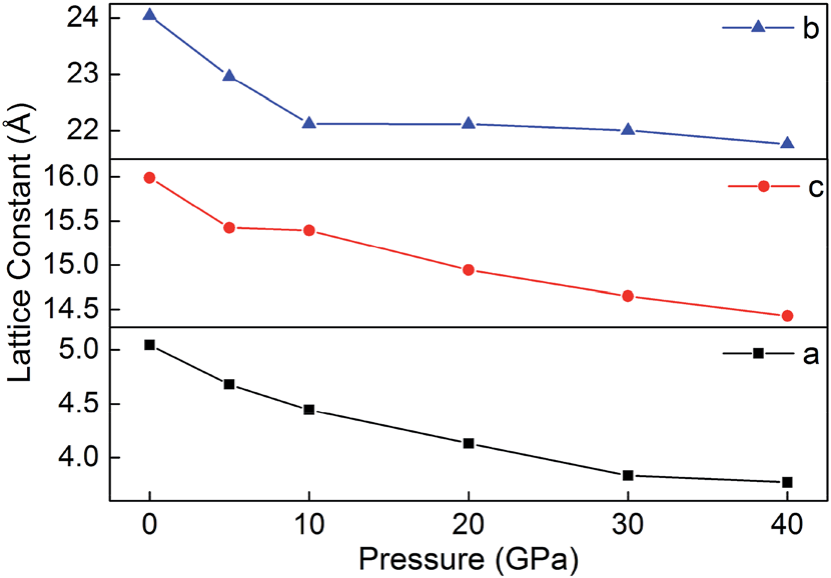
Lattice constants (*a*, *b* and *c*) of B6 in 0–40 GPa.

Fig. 8 displays the relaxed lattice constant (*a*, *b*, and *c*) of B6 in the pressure range of 0–40 GPa. Overall, *a*, *b* and *c* decrease with the increasing pressure. However, with the pressure increasing, the lattice constants *a*, *b* and *c* decrease inconsistently. This may be because B6 is an ionic crystal whose internal ionic bond is non-directional. In addition, during the decline of *a*, *b* and *c*, they do not undergo violent variations, indicating that the ionic crystal is maintained in a stable state under different pressures.

## 4. Conclusion

Four series energetic anions were constructed by replacing nitro group (NO_2_) with trinitromethyl group (C(NO_2_)_3_) or by inserting N-bridging groups (–NH–, –NH–NH–, –N=N–, –N=N(O)–) into the different bistriazole frameworks. The energetic salts were designed based on these anions and hydroxylammonium cation, and their properties were studied by the DFT-B3LYP method. The bistriazole frame containing –C^⊖^(NO_2_)_2_ group, –C(NO_2_)_3_ group and –N=N(O)– linkage is the best anion for increasing the density of title salts. In order to obtain excellent HOFs, choosing the bistriazole frame that contains the deprotonated dinitromethyl group (–C^⊖^(NO_2_)_2_), replacing nitro group (NO_2_) with –C(NO_2_)_3_ group or inserting –N=N(O)– linkage into the framework are good choices. Generally, the bistriazole frame containing deprotonated dinitromethyl group –C^⊖^(NO_2_)_2_, –C(NO_2_)_3_ group or –N=N(O)– linkage is more helpful to obtain good detonation properties due to its larger nitrogen content and better oxygen balance. And one of their corresponding hydroxylammonium salts exhibits better detonation performance (*D* = 10.06 km s^−1^ and *P* = 48.58 GPa) than CL-20 (*D* = 9.54 km s^−1^ and *P* = 43.36 GPa). Moreover, 10 energetic salts not only exhibit excellent energetic properties superior to CL-20, but also have lower sensitivities than CL-20 (*h*_50_ = 13.81 cm). In addition, we rationally selected salt B6 from the 10 salts and predicted the change of its crystal structure under different pressures.

Our design strategy of converting energetic molecules with excellent detonation properties into energetic ions may be a valuable approach to develop novel HEDMs with both excellent performance and low sensitivity.

## Conflicts of interest

There are no conflicts to declare.

## Supplementary Material

RA-009-C9RA05141D-s001
