# Supplementary material for: Theoretical screening of bistriazole-derived energetic salts with high energetic properties and low sensitivity
Source: RSC Adv. 2019 Aug 22;9(45):26442–9. doi: 10.1039/c9ra05141d (PMC9070392; doi:10.1039/c9ra05141d)
Supplement: RA-009-C9RA05141D-s001 [file RA-009-C9RA05141D-s001.pdf]

Electronic Supplementary Material (ESI) for RSC Advances.
This journal is © The Royal Society of Chemistry 2019

Supporting Information

Theoretical screening of bistriazole-derived energetic salts with high energetic properties and low sensitivity

Xiao-Hong Li [1], Cong Zhang [2], Xue-Hai Ju [2*]

[1] College of Physics and Engineering, Henan University of Science and Technology, Luoyang, 471003, China;

[2] Key Laboratory of Soft Chemistry and Functional Materials of MOE, School of Chemical Engineering, Nanjing University of Science and Technology, Nanjing 210094, P. R. China.

Contents

Scheme S1. Born−Haber energy cycle for the formation of the salts
Scheme S2. Isodesmic reactions for the anions
Scheme S3. Born−Haber cycle for the formation of the salts
Table S1. Volumes (cm^3 mol^{-1}) and densities (g cm^{-3}) of the salts
Table S2. Calculated and experimental gas-phase heats of formation (kJ mol^{-1}) for small molecules and ions at 298 K
Table S3. The HOFs ((kJ mol^{-1}) of cage anions, ammonium-based cation, and their salts and lattice energies of these salts
Table S4. Predicted entropies (S_{salt}), entropies of reaction (ΔS_{rxn}), enthalpies of reaction (ΔH_{rxn}), and free energies of reaction (ΔG_{rxn}) of the salts
Figure S1. Optimized crystal structure of the **B6**
Figure S2. Optimized structures of the related anions and cations

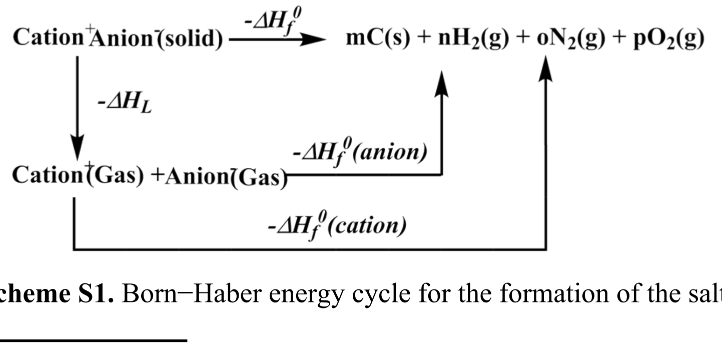

Scheme S1. Born−Haber energy cycle for the formation of the salts

* Corresponding author: email: xhju@njust.edu.cn

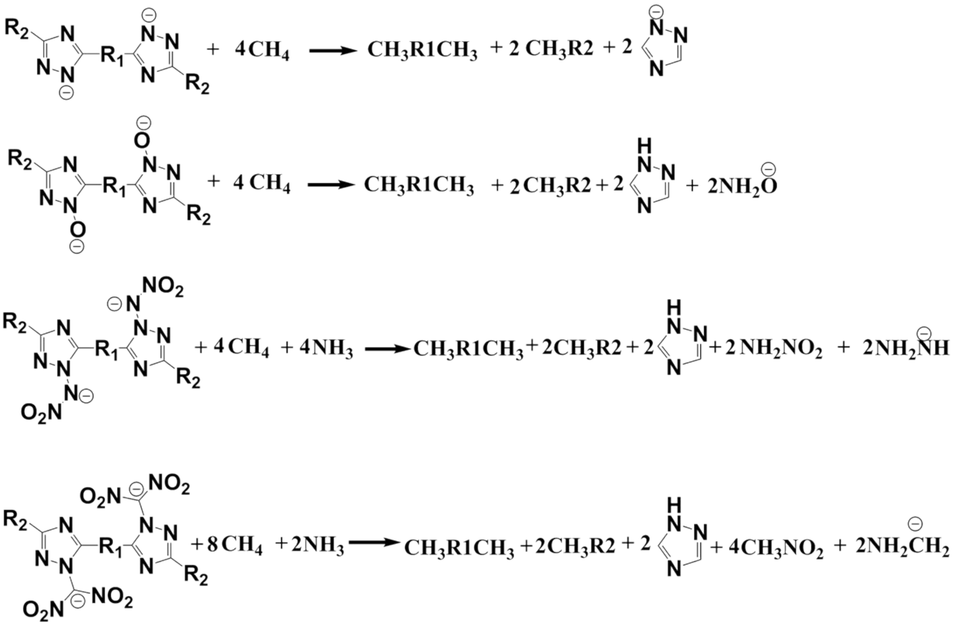

Scheme S2. Isodesmic reactions for the anions.

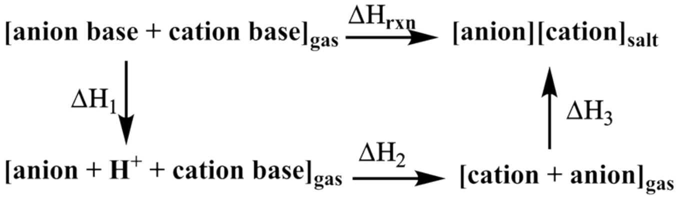

Scheme S3. Born−Haber cycle for the formation of the salts

Table S1. Volumes (cm^3 mol^{-1}) and densities (g cm^{-3}) of the salts

Salts	V	A_s^+	V_s^+	A_s^-	V_s^-	ρ
A1	163.05	61.68	152.51	226.28	–155.43	1.71
A2	171.32	61.68	152.51	242.08	–149.09	1.73
A3	181.02	61.68	152.51	256.75	–142.99	1.73
A4	178.56	61.68	152.51	253.89	–144.84	1.74
A5	184.77	61.68	152.51	261.83	–143.45	1.77
A6	261.68	61.68	152.51	359.28	–128.75	1.92 (1.97) [a]
A7	269.1	61.68	152.51	374.25	–124.67	1.93
A8	281.83	61.68	152.51	390.81	–120.27	1.91
A9	276.56	61.68	152.51	386.7	–121.91	1.93
A10	285.51	61.68	152.51	391.61	–125.58	1.93
B1	176.44	61.68	152.51	242.56	–150.35	1.77 (1.9) [b]
B2	185.76	61.68	152.51	261.73	–144.35	1.78
B3	194.26	61.68	152.51	275.96	–138.11	1.78
B4	188.57	61.68	152.51	272.3	–140.87	1.82
B5	197.62	61.68	152.51	278.92	–139.28	1.82
B6	271.38	61.68	152.51	376	–125.69	1.98 (1.99) [c]
B7	282.51	61.68	152.51	394.76	–122.14	1.96

B8	293.52	61.68	152.51	417.69	−115.54	1.95
B9	289.18	61.68	152.51	404.98	−119.51	1.96
B10	291.16	61.68	152.51	405.19	−123.93	2.00
C1	213.38	61.68	152.51	315.31	−135.61	1.90 (1.86) [d]
C2	223.08	61.68	152.51	325.43	−131.88	1.90
C3	238.1	61.68	152.51	344.52	−127.2	1.86
C4	238.21	61.68	152.51	342.94	−128.36	1.85
C5	238.24	61.68	152.51	349.69	−126.17	1.91
C6	323.85	61.68	152.51	446.43	−119.36	1.96
C7	331.41	61.68	152.51	447.13	−117.51	1.97
C8	334.08	61.68	152.51	475	−112.62	2.00
C9	337.31	61.68	152.51	476.92	−112.18	1.97
C10	341.41	61.68	152.51	482.45	−111.35	2.00
D1	259.48	61.68	152.51	366.5	−127.63	1.94 (1.86) [e]
D2	271.2	61.68	152.51	385.15	−122.44	1.92
D3	281.28	61.68	152.51	401.41	−117.95	1.91
D4	277.28	61.68	152.51	401.87	−119.11	1.93
D5	281.85	61.68	152.51	399.77	−120.9	1.95
D6	355.11	61.68	152.51	499.74	−114.63	2.05
D7	371.82	61.68	152.51	511.85	−111.36	2.01
D8	380.91	61.68	152.51	531.56	−106.36	2.01
D9	381.98	61.68	152.51	535.38	−107.19	2.00
D10	375.36	61.68	152.51	534.93	−107.5	2.07

[a] The value is from Ref[1] ; [b] The value is from Ref[2]; [c] At the B3LYP/aug-cc-pVTZ level; [d] The value is from Ref[3]; [e] The value is from Ref[4].

Table S2. Calculated and experimental gas-phase heats of formation (kJ mol^{-1}) for small molecules and ions at 298 K

Molecules/ions	ΔH_f^o(calculated)	ΔH_f^o(literature)
CH_4	−73.6	−74.4[a]
NH_3	−45.3	−46.1[a]
CH_3NO_2	−85.9	−80.8[a]
$CH_3C(NO_2)_3$	−23.2	
NH_2NO_2	−3.9	−3.2[a]
CH_3CH_3	−84	−87[a]
CH_3NHCH_3	−18.8	−19.1[a]
$CH_3NHNHCH_3$	92.2	93.5[a]
$CH_3N{=}NCH_3$	151.5	153.2[a]
$CH_3N{=}N(O)CH_3$	−40.16	
1,2,4-triazole	192.9	192.9[a]
H^+		1536.2
NH_2O^-	37.21[b]	36.5[c]
NH_2NH^-	223.92[b]	225.2[c]
$NH_2CH_2^-$	196.11[b]	193.4[c]

1,2,4-triazolide	102.8[b]

[a] The values were taken from Ref[5]. [b] The values were predicted by protonation reactions: $NH_2O^- + H^+ = NH_2OH$, $NH_2NH^- + H^+ = NH_2NH_2$, $NH_2CH_2^- + H^+ = NH_2CH_3$, 1,2,4-triazolide + H^+ = 1,2,4-triazole. [c] The values were taken from Ref[6].

Table S3. The HOFs ((kJ mol^{-1}) of cage anions, ammonium-based cation, and their salts and lattice energies of these salts

**Salts**	**ΔH_f^o (cation)**	**ΔH_f^o (anion)**	**Lattice energy**	**ΔH_f^o (salt)**
A1	678.80	258.95	1338.87	277.69
A2	678.80	261.65	1317.99	301.26
A3	678.80	344.77	1294.99	407.39
A4	678.80	431.29	1300.50	488.40
A5	678.80	301.91	1285.45	374.06
A6	678.80	279.11	1140.78	495.93
A7	678.80	282.31	1129.78	510.13
A8	678.80	366.48	1112.20	611.88
A9	678.80	464.94	1119.14	703.40
A10	678.80	376.16	1106.56	627.20
B1	678.80	110.71	1304.25	164.06 (213)[a]
B2	678.80	82.48	1282.98	157.10
B3	678.80	158.59	1264.41	251.78
B4	678.80	262.29	1276.13	343.76
B5	678.80	153.54	1256.37	254.77
B6	678.80	148.22	1125.68	380.13 (358.61)[b]
B7	678.80	126.47	1110.35	373.72
B8	678.80	210.63	1095.96	472.27
B9	678.80	312.36	1101.29	568.67
B10	678.80	237.38	1097.89	497.09
C1	678.80	288.21	1223.49	422.31 (535)[c]
C2	678.80	263.28	1205.47	415.41
C3	678.80	362.19	1179.92	539.87
C4	678.80	512.60	1179.85	690.35
C5	678.80	376.81	1178.78	555.63
C6	678.80	348.34	1058.01	647.93
C7	678.80	463.48	1049.23	771.85
C8	678.80	408.72	1045.97	720.35
C9	678.80	554.25	1042.70	869.15
C10	678.80	448.45	1037.82	768.22
D1	678.80	-30.40	1144.01	183.18 (360)[d]
D2	678.80	-51.50	1127.06	179.03
D3	678.80	35.25	1113.04	279.81
D4	678.80	175.93	1118.36	415.17
D5	678.80	98.03	1111.43	344.20
D6	678.80	40.85	1022.38	376.07

D7	678.80	39.46	1006.09	390.97
D8	678.80	104.95	997.44	465.11
D9	678.80	251.09	996.56	612.13
D10	678.80	174.03	1001.92	529.71

[a] The value is from Ref[2]; [b] At the B3LYP/aug-cc-pVTZ level; [c] The value is from Ref[3]; [d] The value is from Ref[4].

Table S4. Predicted entropies (S_{salt}), entropies of reaction ($\triangle S_{rxn}$), enthalpies of reaction ($\triangle H_{rxn}$), and free energies of reaction ($\triangle G_{rxn}$) of the salts.

Anion	S_{salts}	ΔS_{rxn}	ΔH_{rxn}	ΔG_{rxn}
A1	276.19	-691.82	-432.22	-226.06
A2	285.54	-693.82	-377.04	-170.28
A3	296.46	-731.87	-400.48	-182.39
A4	293.78	-708.75	-437.10	-225.89
A5	301.19	-715.84	-429.20	-215.88
A6	391.20	-860.75	-309.18	-52.68
A7	399.75	-872.18	-263.69	-3.78
A8	414.02	-885.93	-279.08	-15.08
A9	408.29	-880.03	-318.55	-56.30
A10	418.77	-881.39	-317.63	-54.97
B1	291.98	-695.01	-464.75	-257.63
B2	302.44	-715.09	-438.67	-225.57
B3	312.09	-761.65	-466.74	-239.77
B4	305.94	-742.85	-494.29	-272.92
B5	316.42	-751.01	-485.64	-261.84
B6	403.00	-883.34	-348.31	-85.08
B7	415.57	-899.15	-323.99	-56.04
B8	427.93	-928.01	-340.17	-63.63
B9	423.28	-914.07	-368.50	-96.11
B10	426.23	-911.47	-358.74	-87.12
C1	335.20	-788.18	-443.03	-208.16
C2	346.27	-807.48	-445.82	-205.19
C3	362.99	-821.64	-415.18	-170.33
C4	363.04	-792.74	-433.93	-197.69
C5	363.76	-828.79	-452.83	-205.85
C6	463.35	-959.30	-319.71	-33.83
C7	472.18	-965.13	-334.90	-47.29
C8	475.53	-986.16	-322.10	-28.23
C9	478.91	-983.29	-337.52	-44.50
C10	484.04	-996.58	-339.82	-42.83
D1	388.74	-868.05	-428.31	-169.63
D2	401.90	-873.65	-441.33	-180.98
D3	413.32	-919.82	-462.59	-188.49
D4	408.93	-898.29	-441.32	-173.63
D5	414.67	-915.87	-435.54	-162.61

D6	500.83	-1033.40	-347.35	-39.40
D7	517.25	-1056.83	-351.61	-36.68
D8	529.85	-1069.37	-351.33	-32.65
D9	530.92	-1067.02	-342.02	-24.05
D10	524.45	-1091.43	-350.23	-24.98

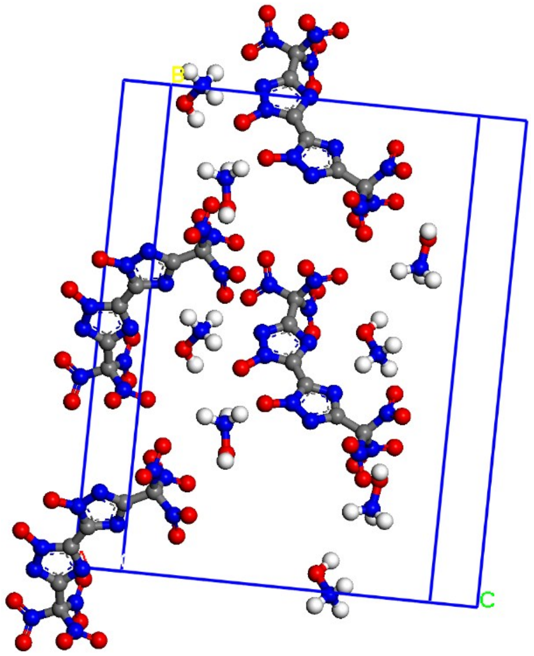

Figure S1. Optimized crystal structure of the **B6**

Anion A1

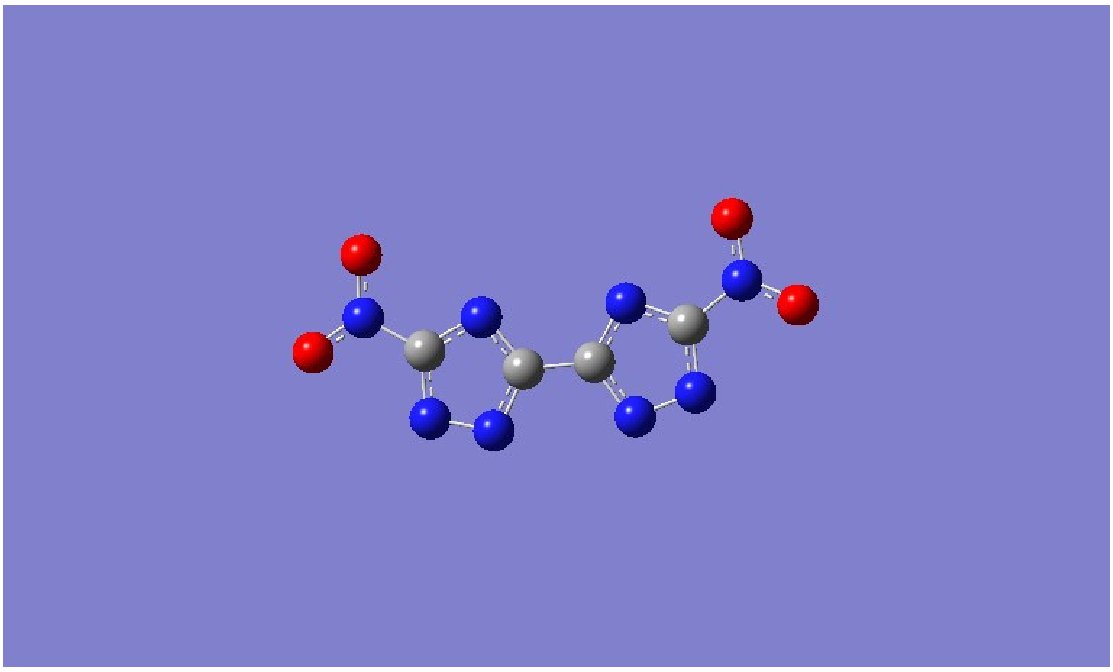

Anion A2

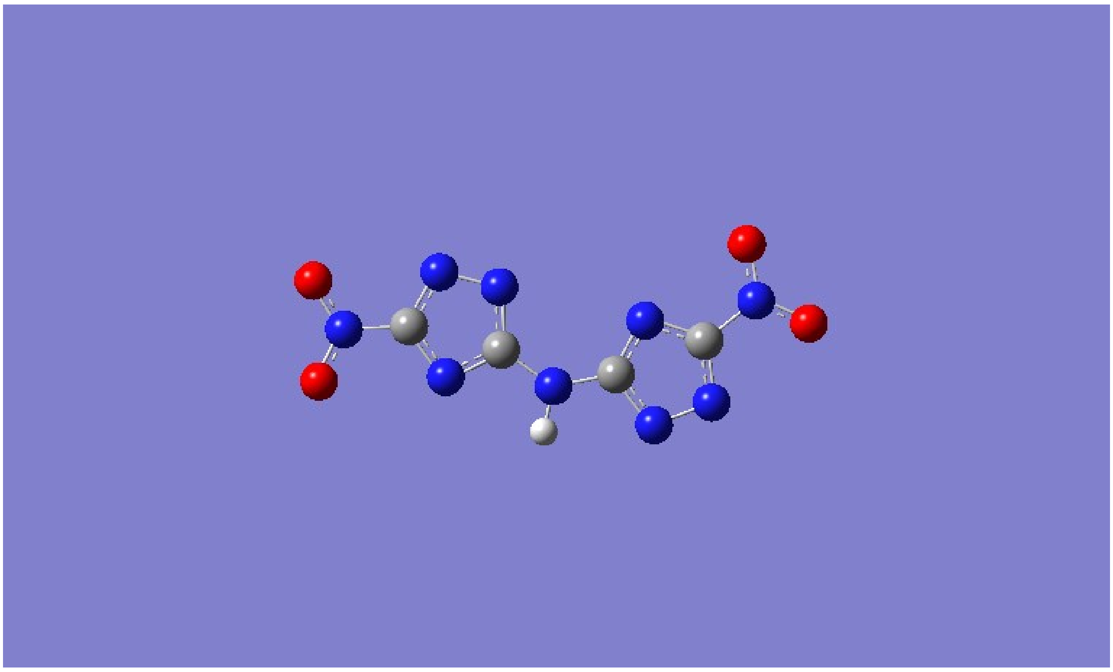

Anion A3

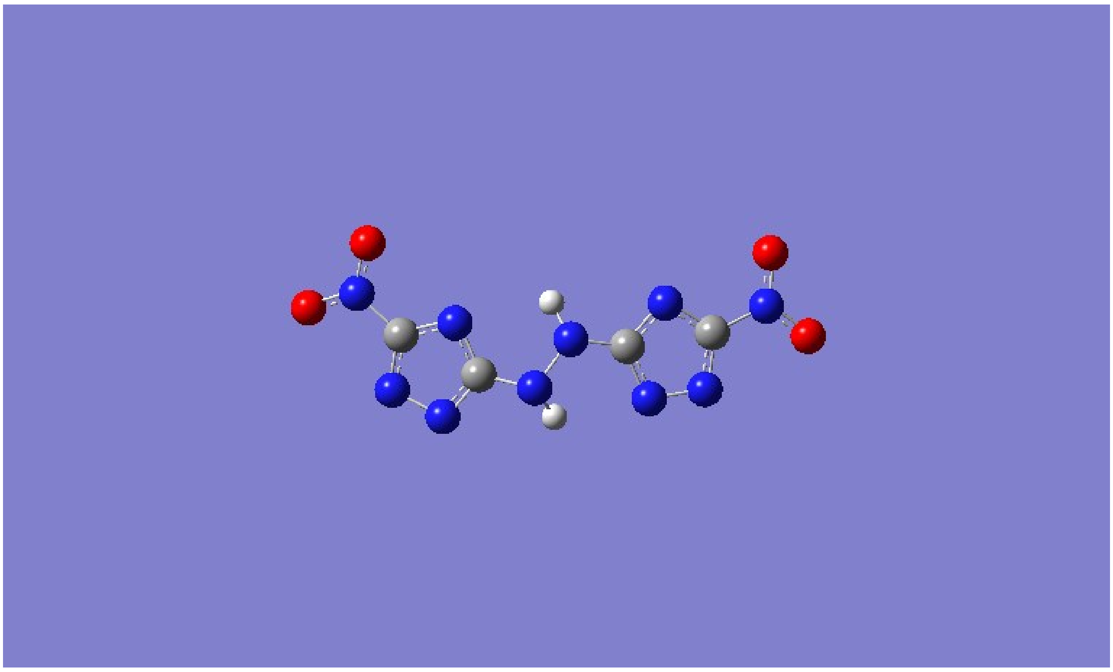

Anion A4

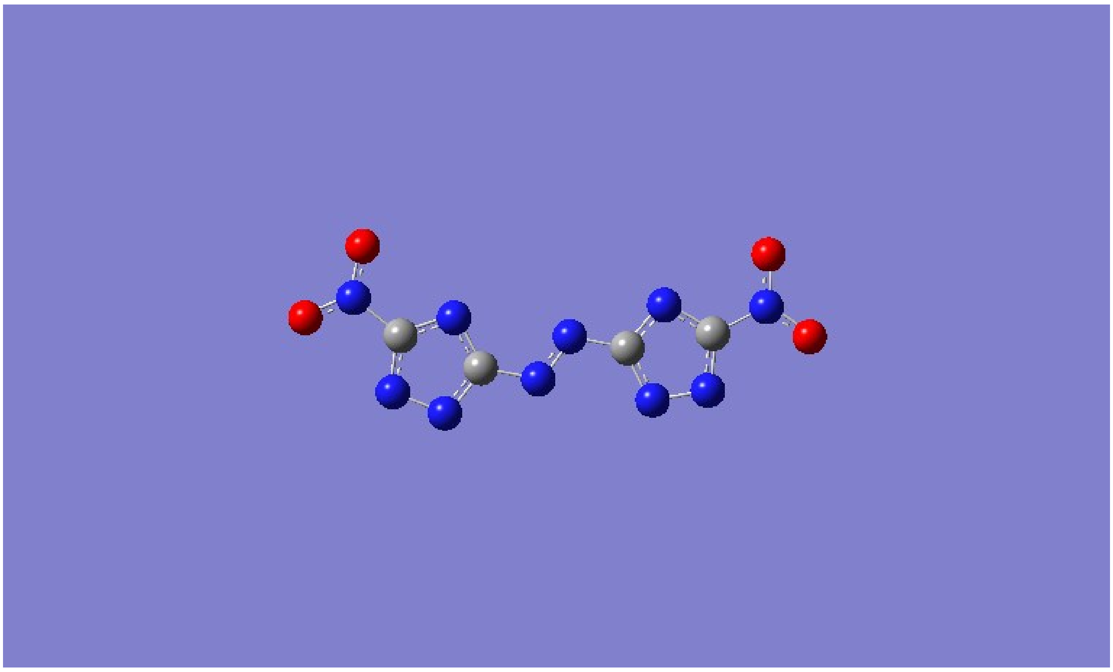

Anion A5

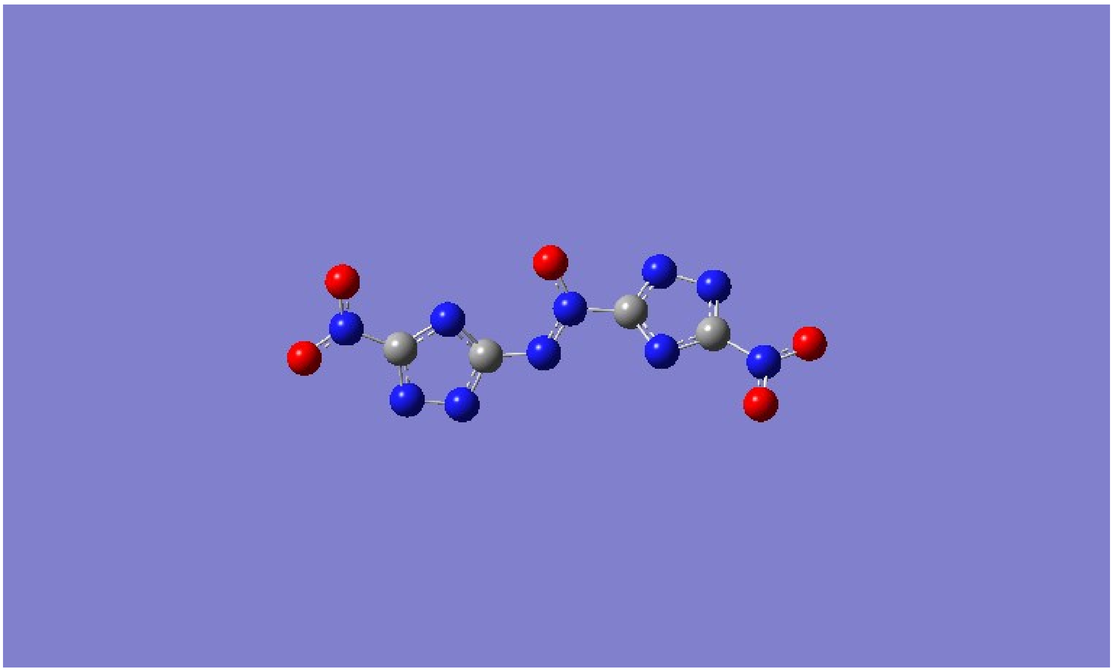

Anion A6

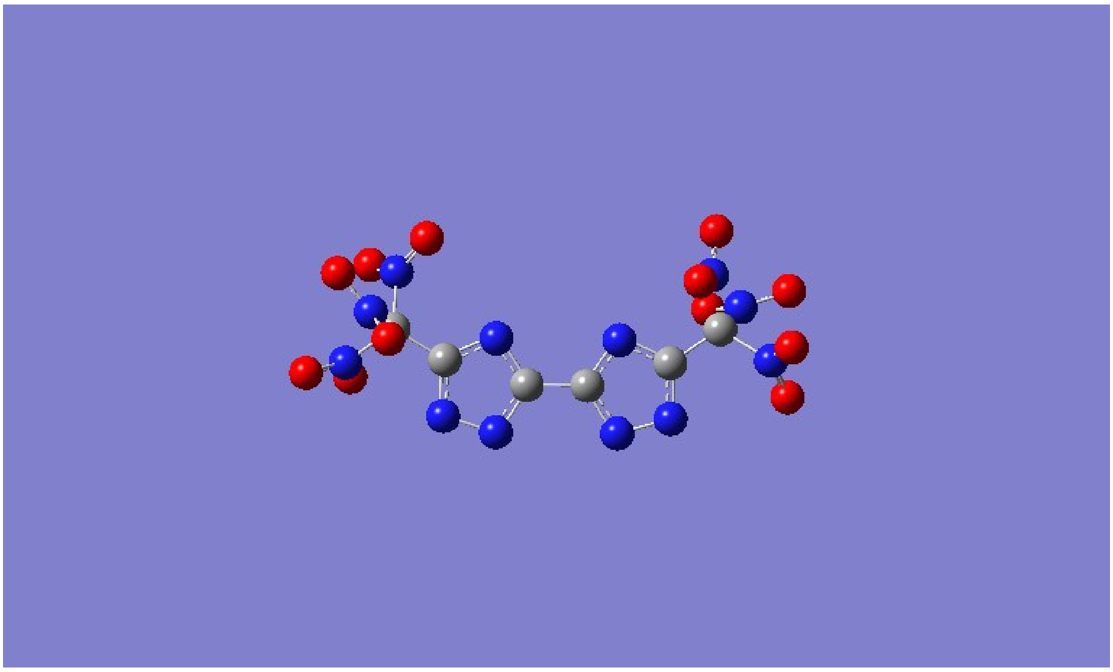

Anion A7

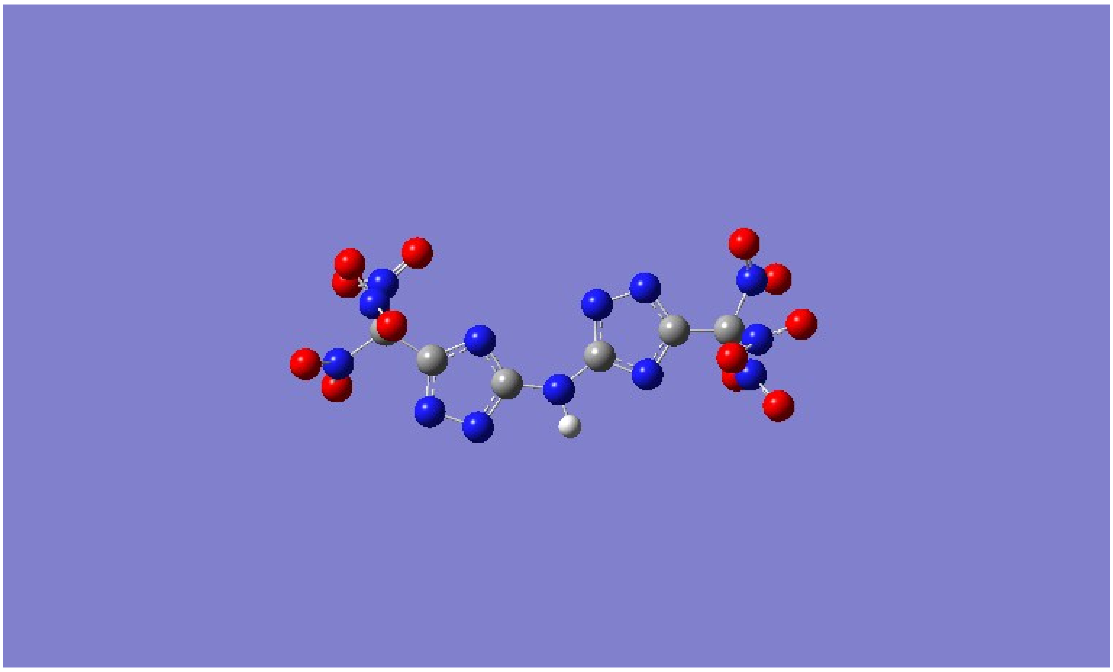

Anion A8

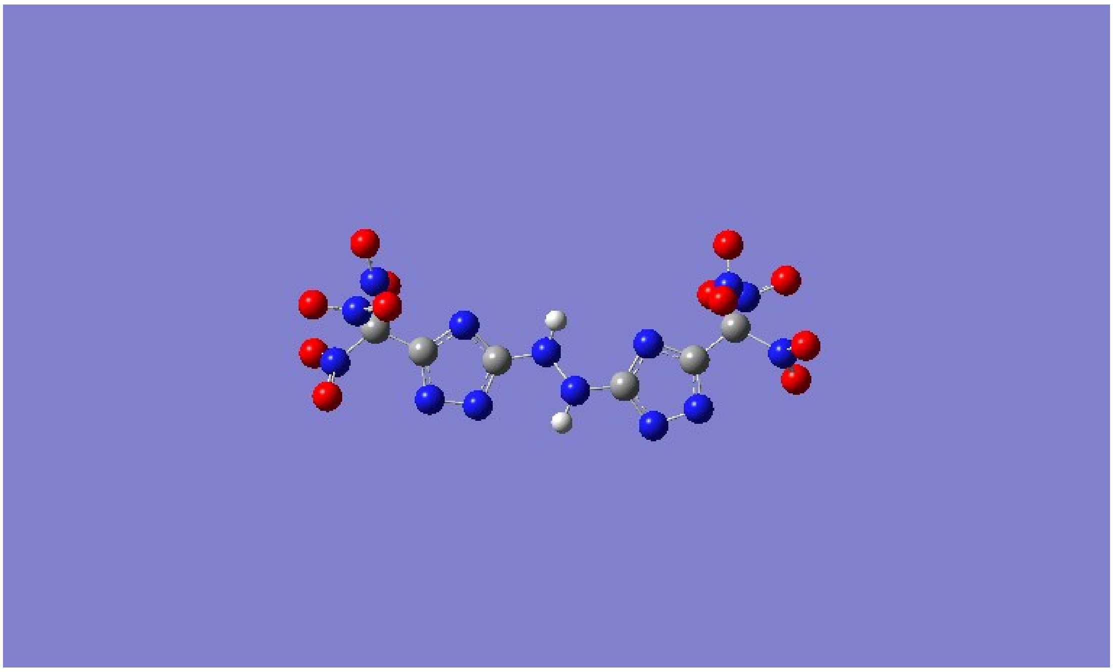

Anion A9

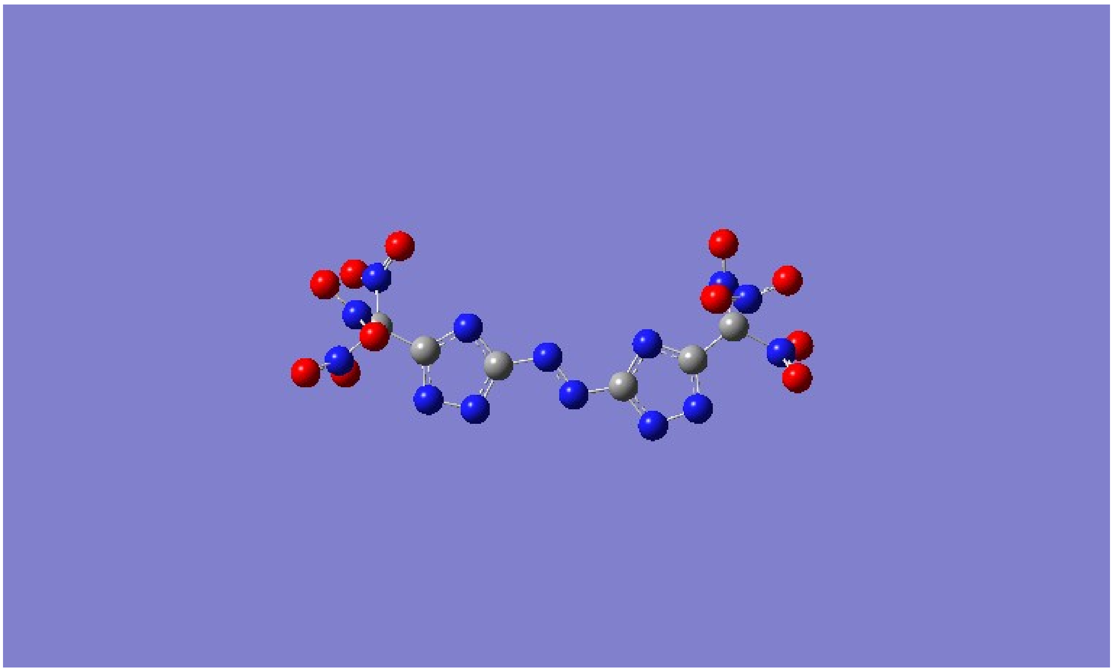

Anion A10

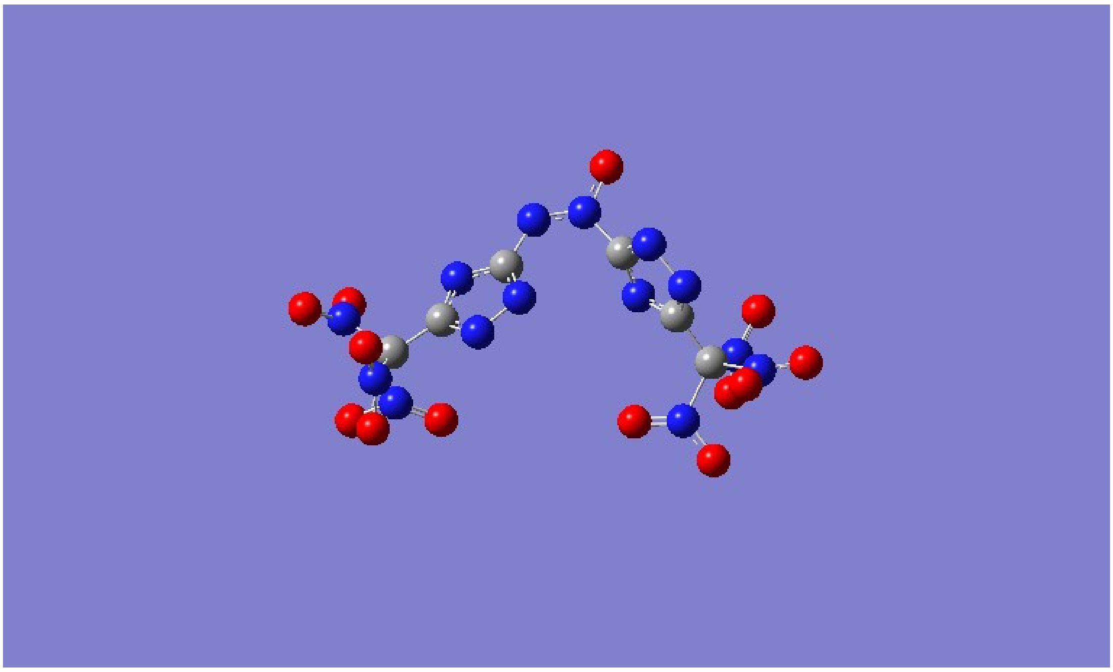

Anion B1

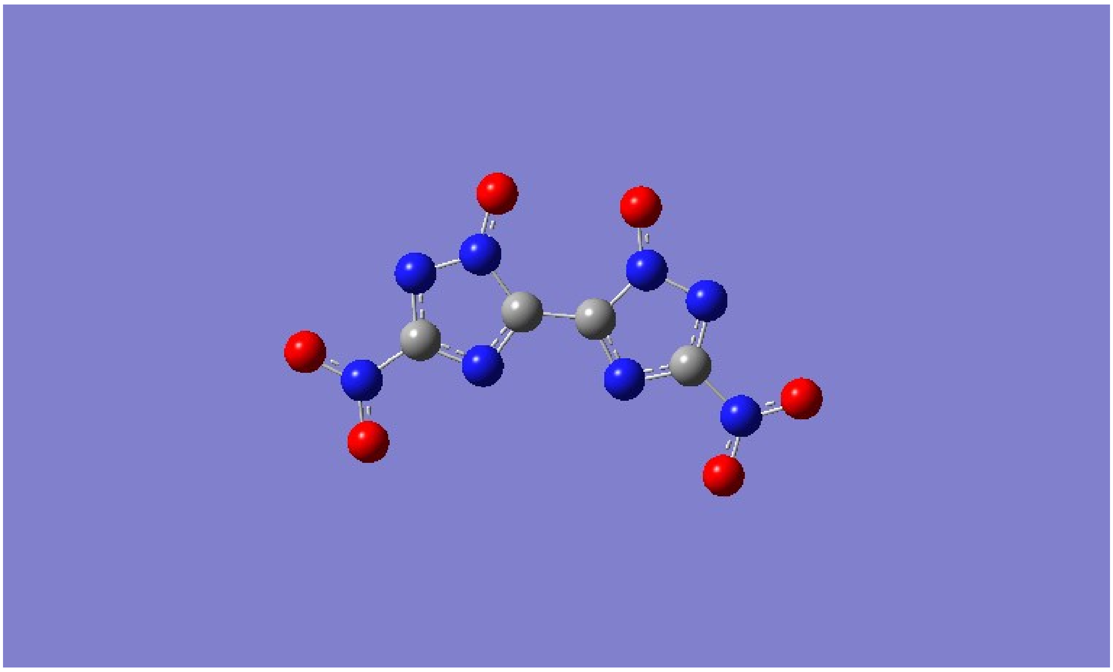

Anion B2

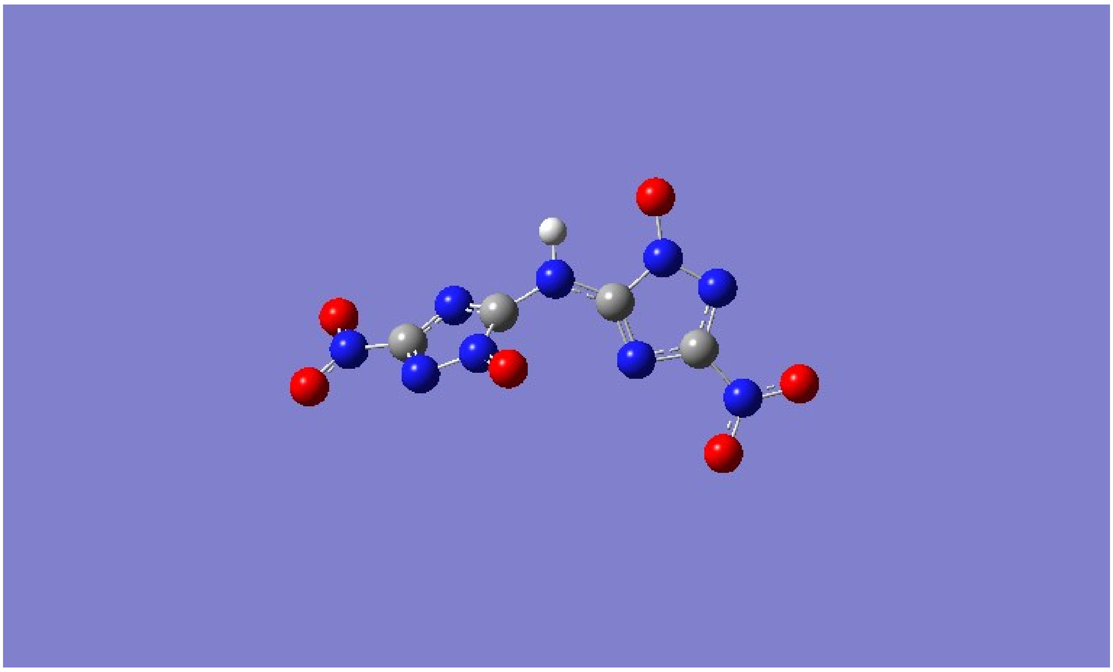

Anion B3

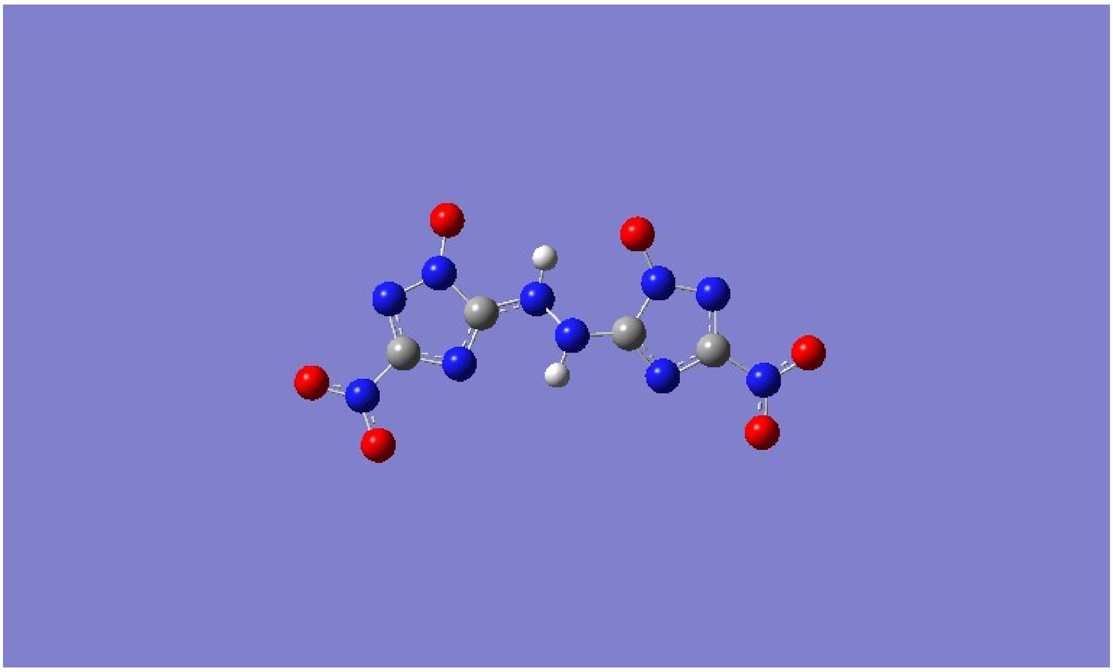

Anion B4

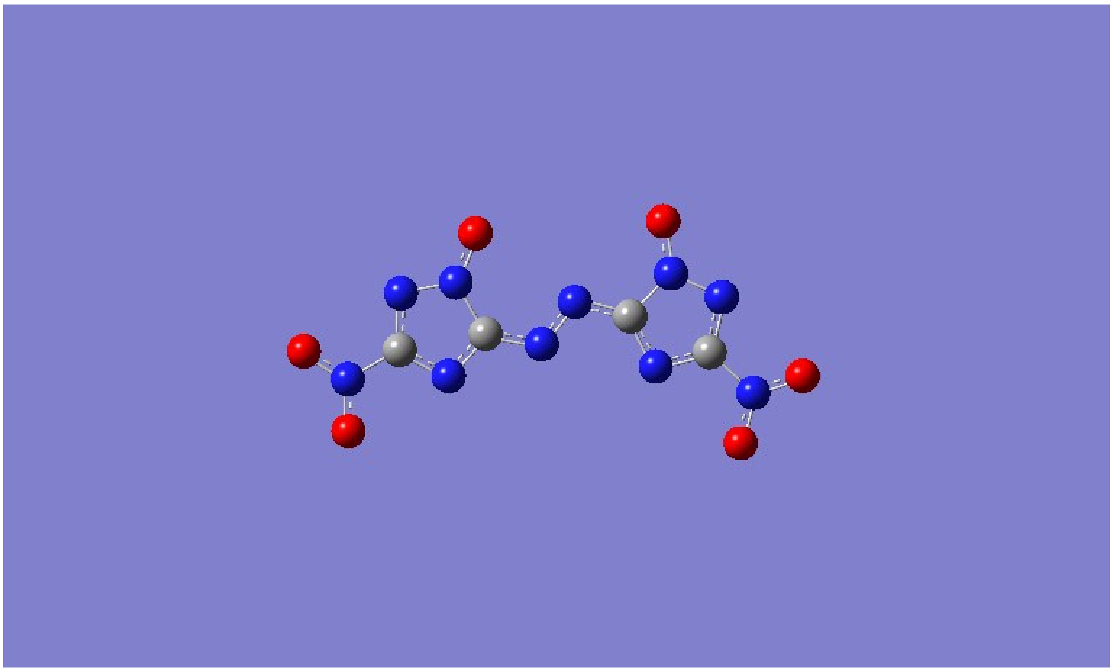

Anion B5

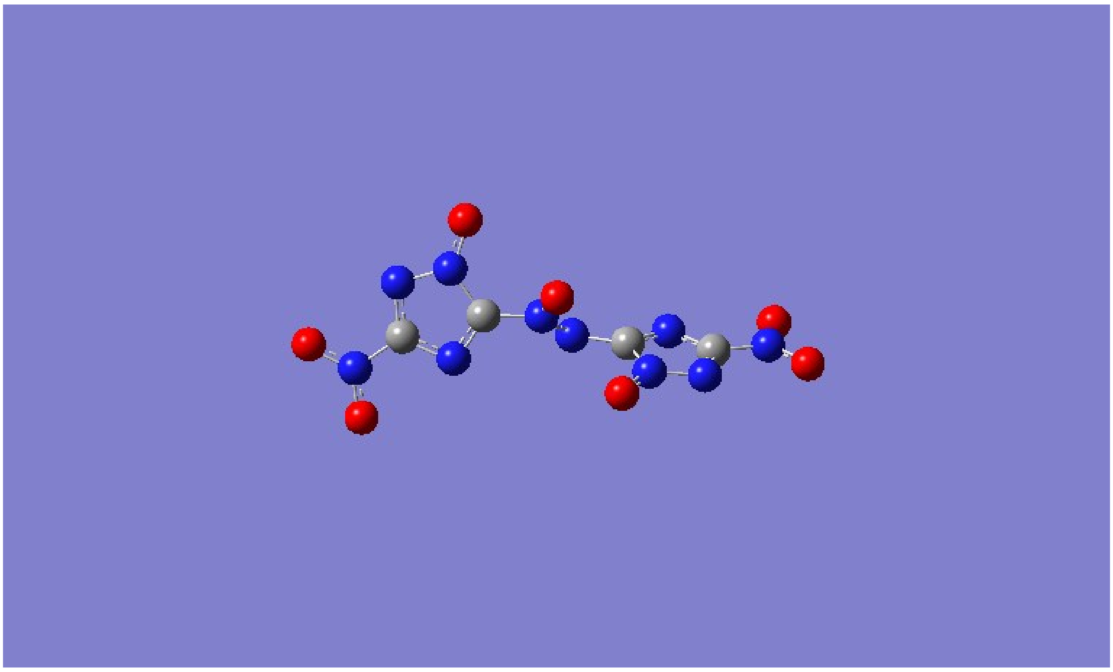

Anion B6

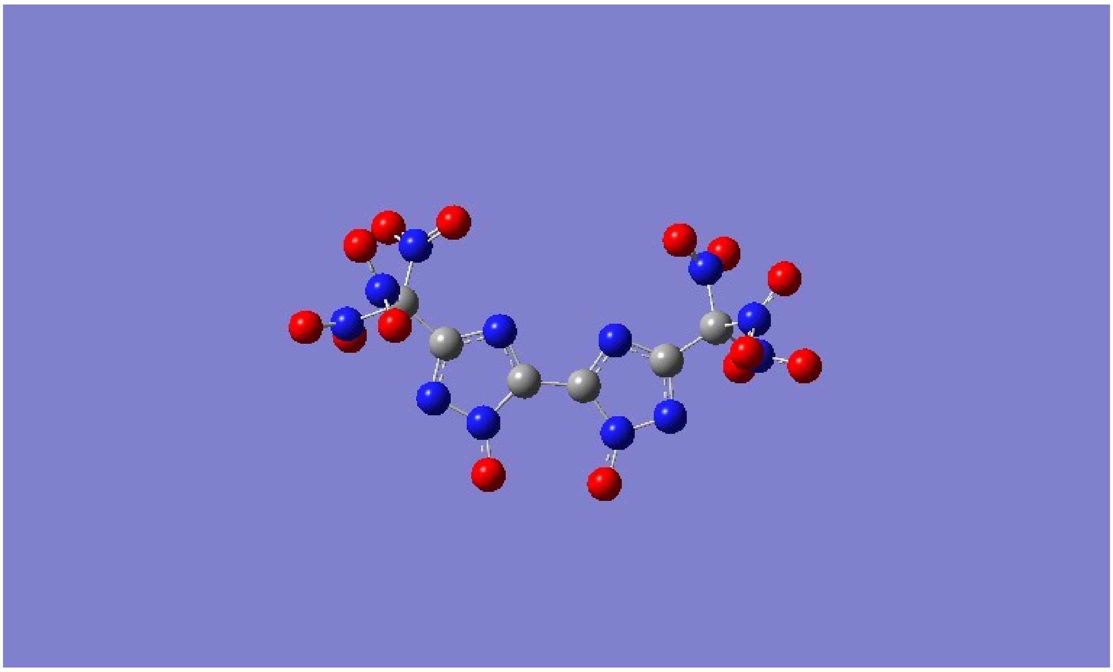

Anion B7

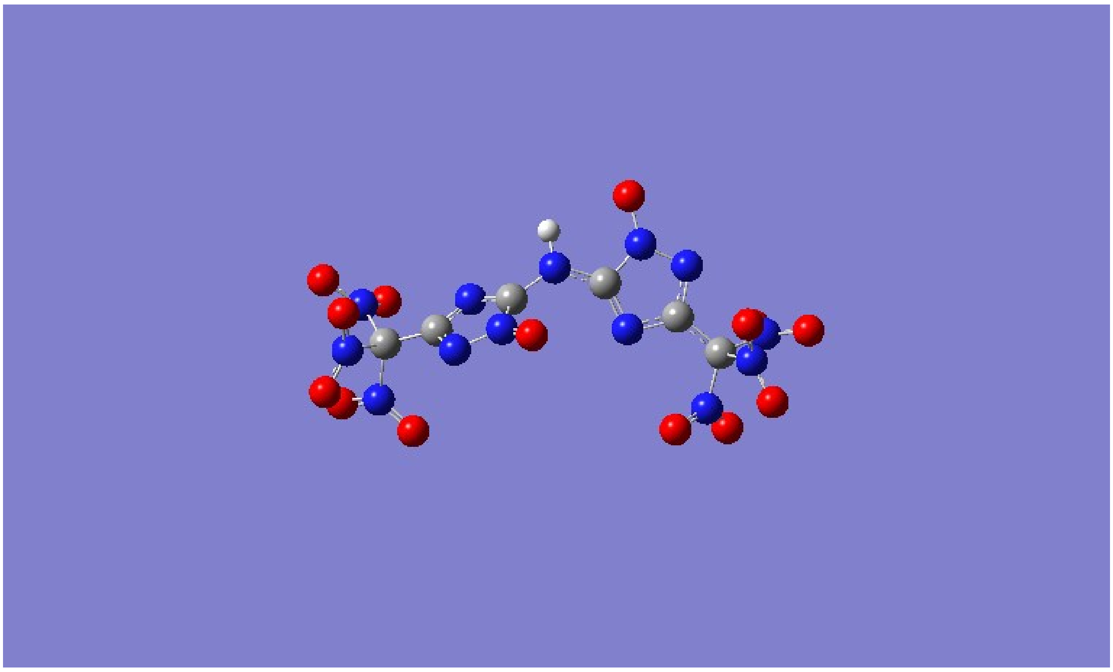

Anion B8

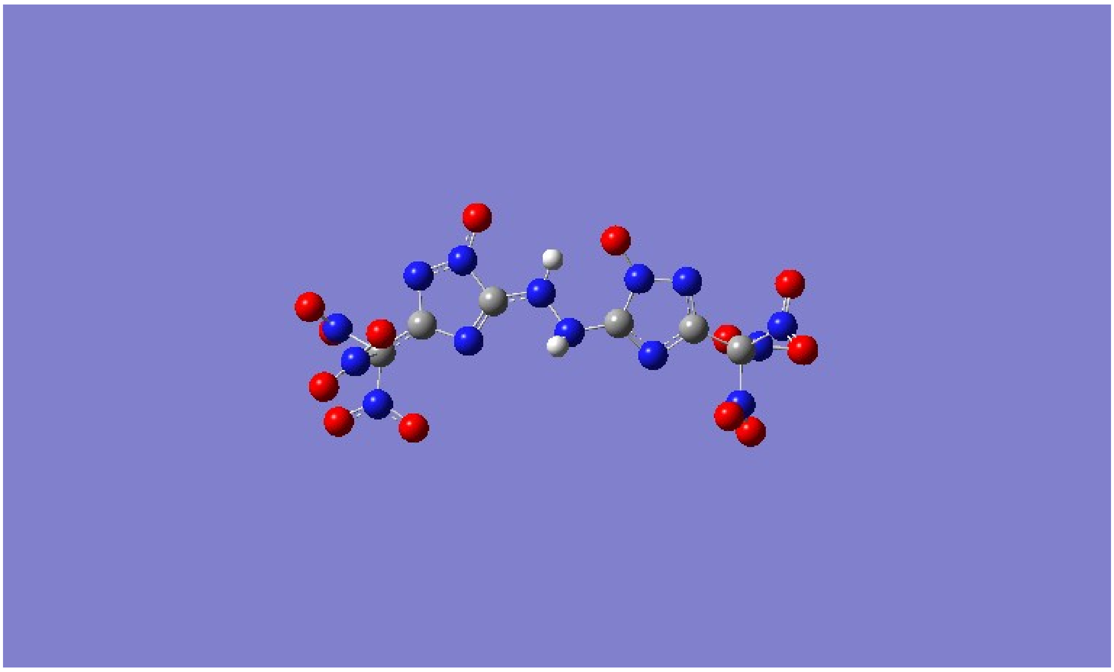

Anion B9

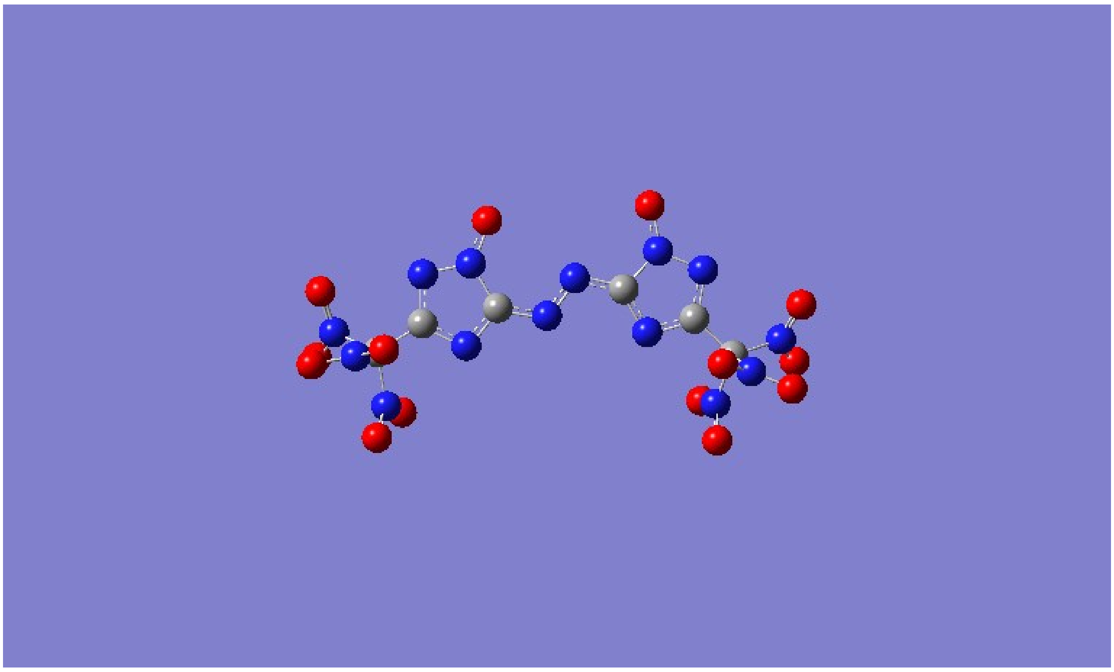

Anion B10

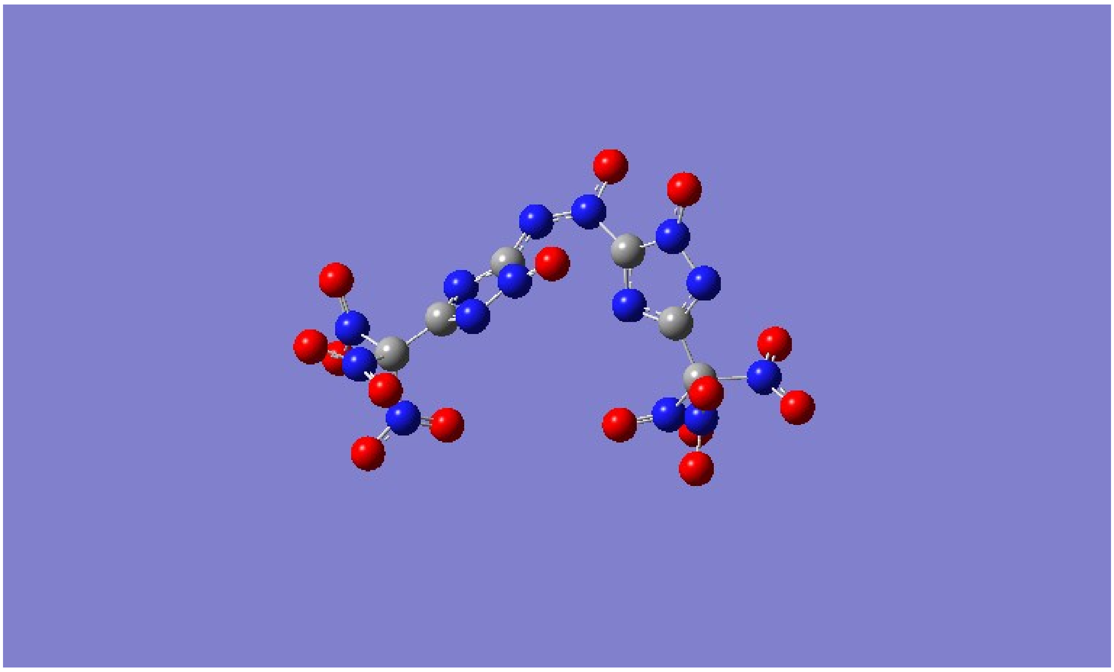

Anion C1

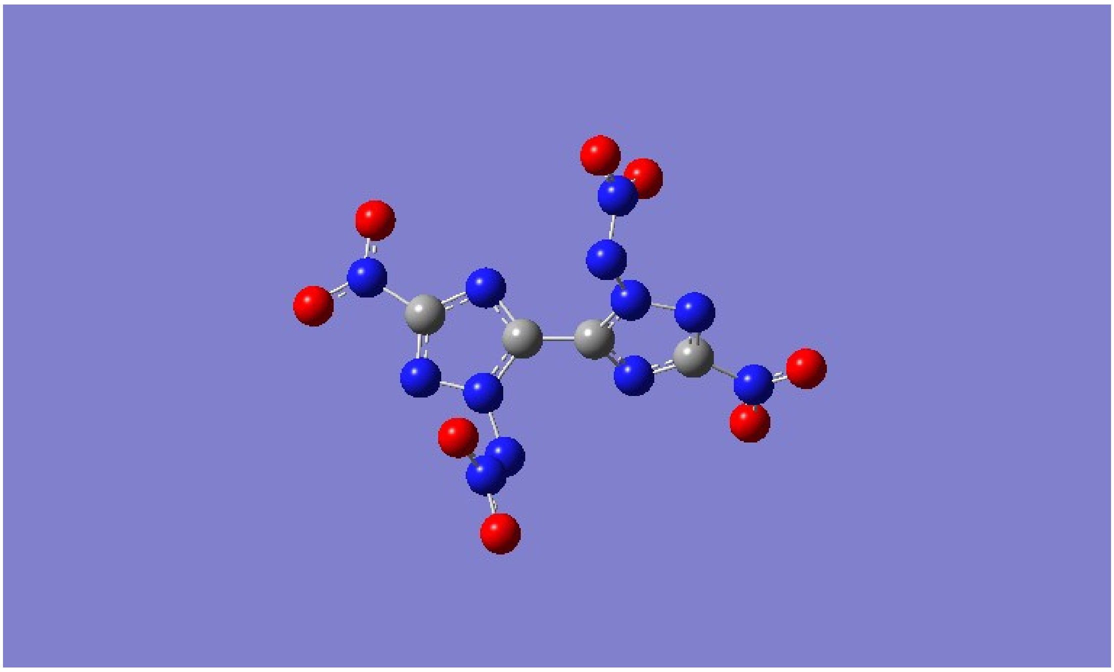

Anion C2

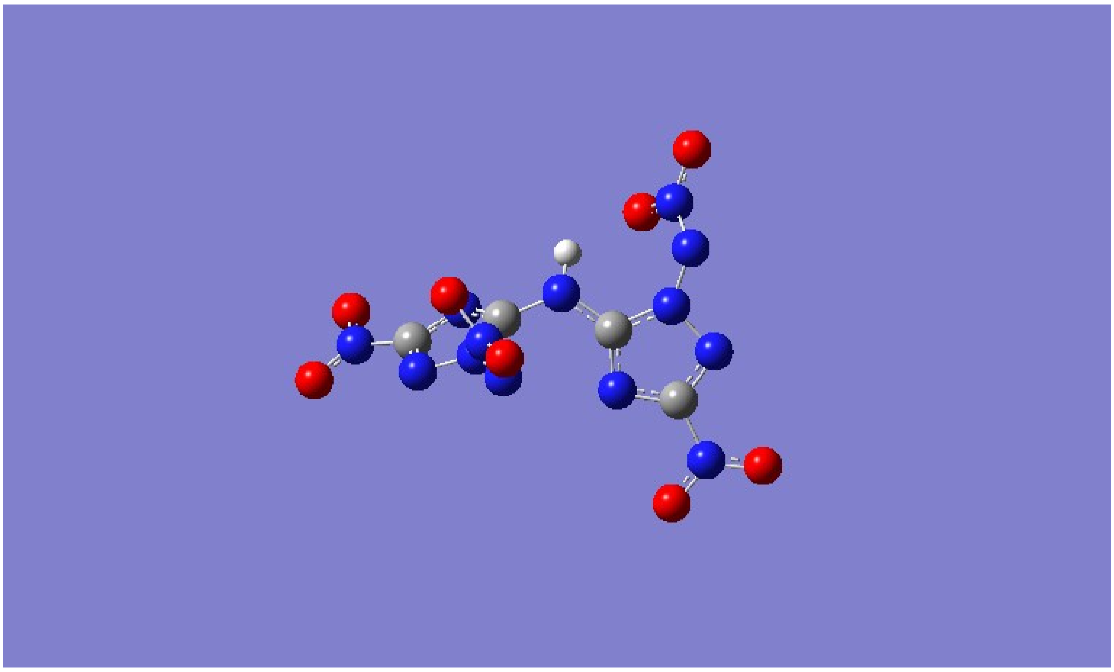

Anion C3

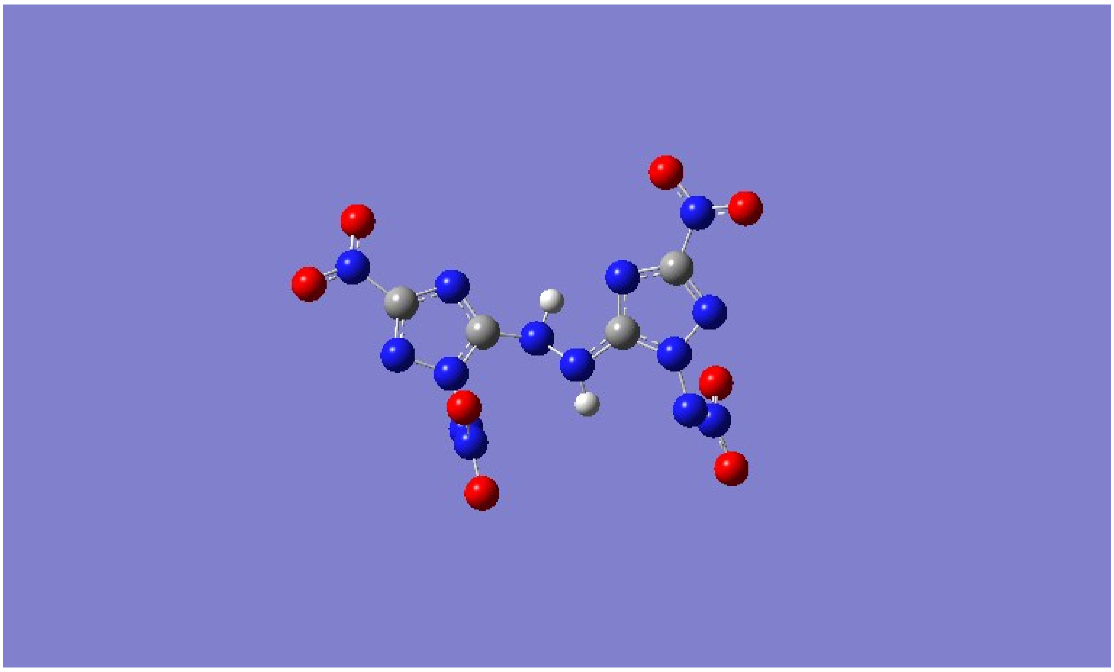

Anion C4

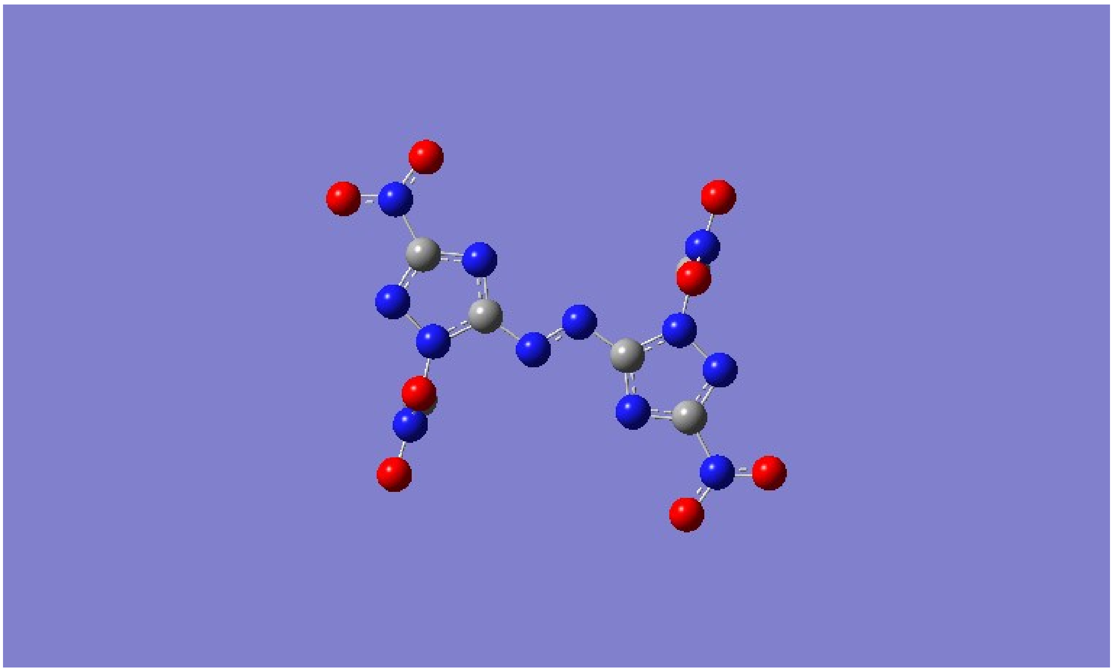

Anion C5

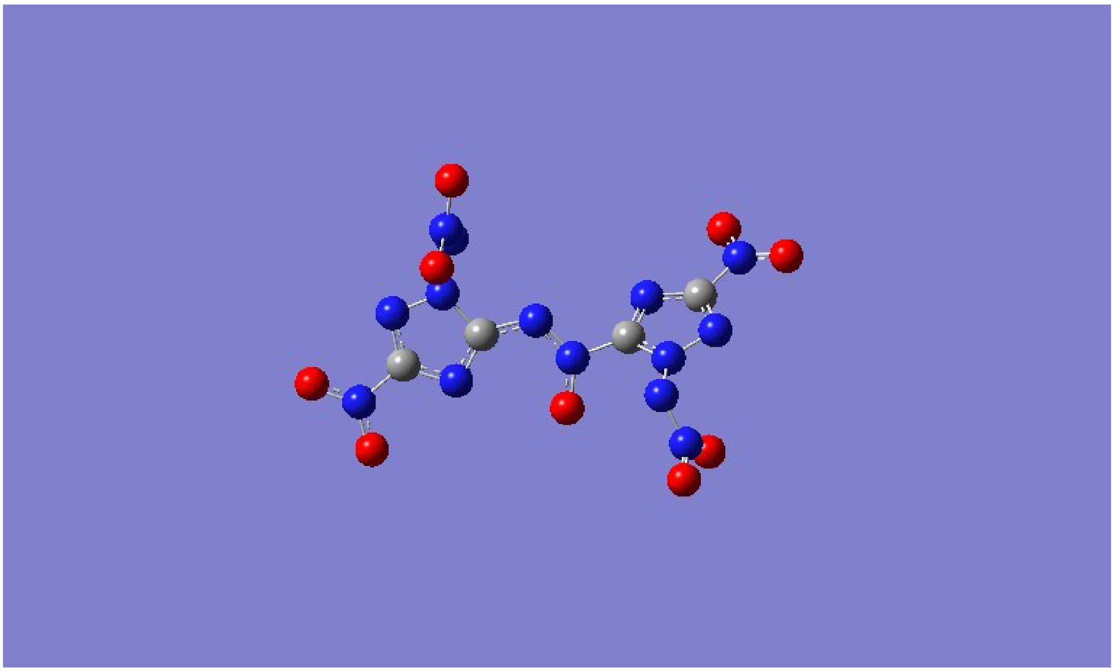

Anion C6

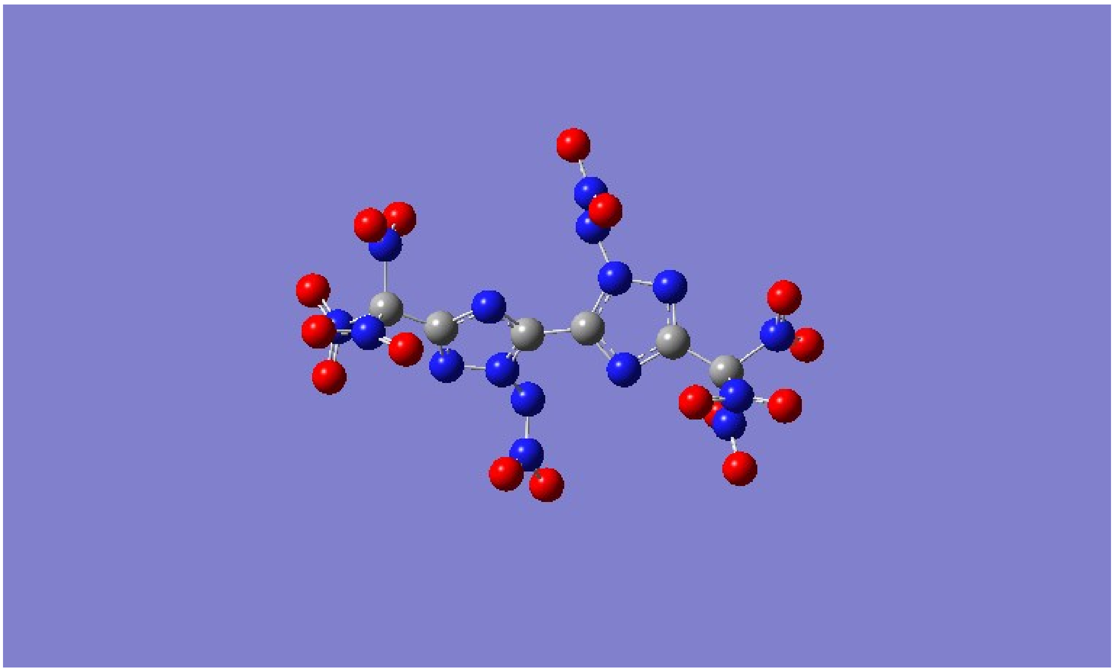

Anion C7

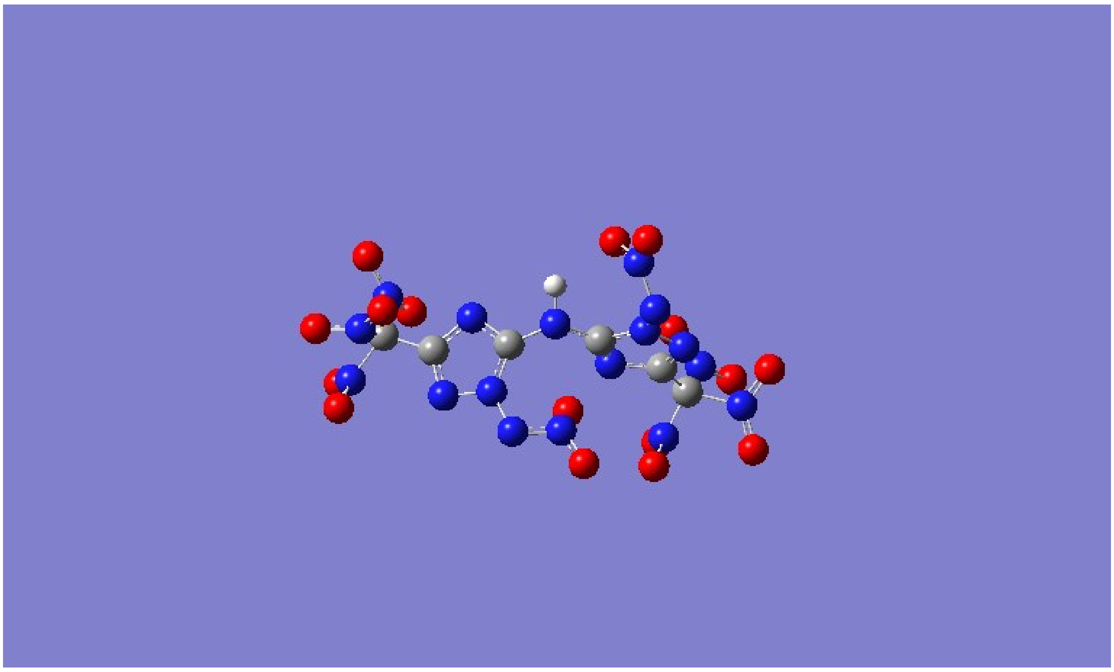

Anion C8

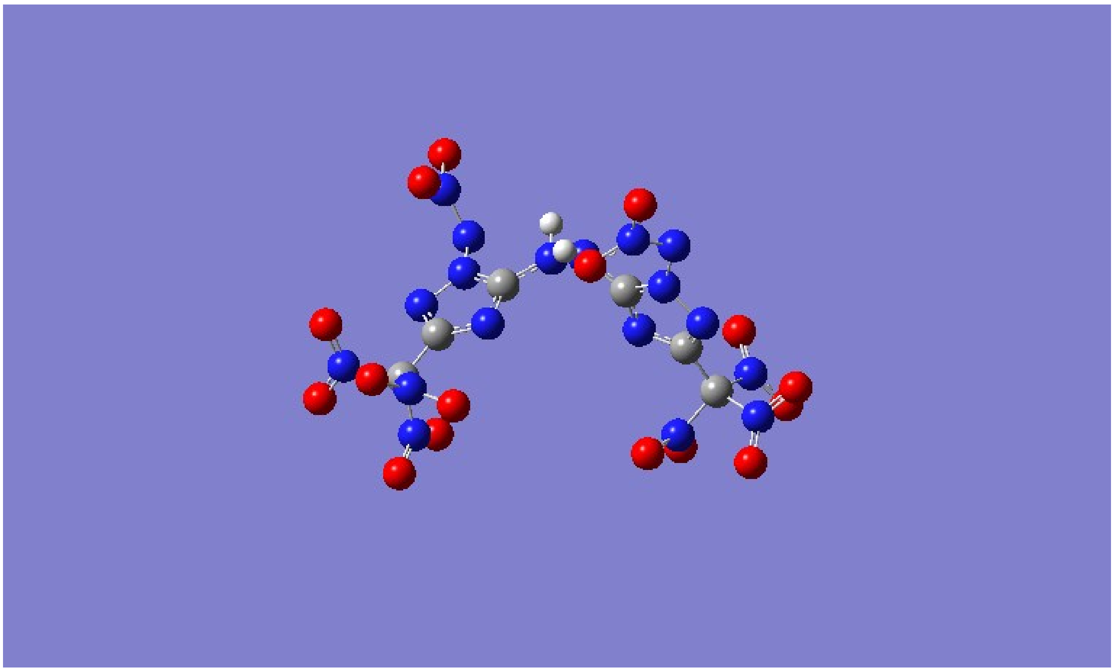

Anion C9

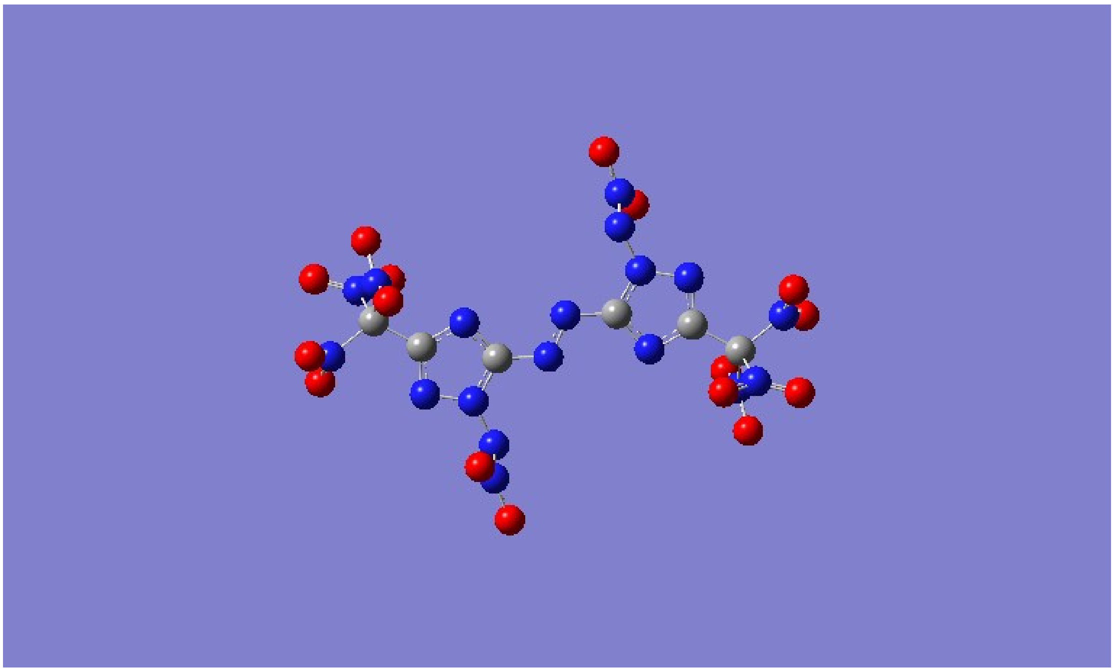

Anion C10

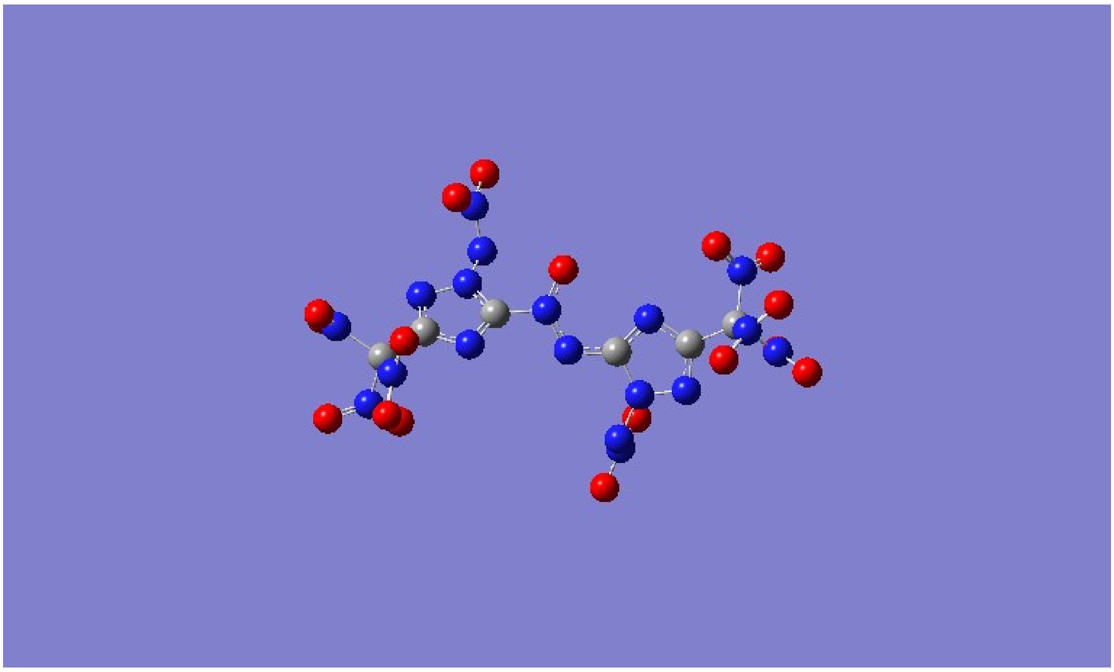

Anion D1

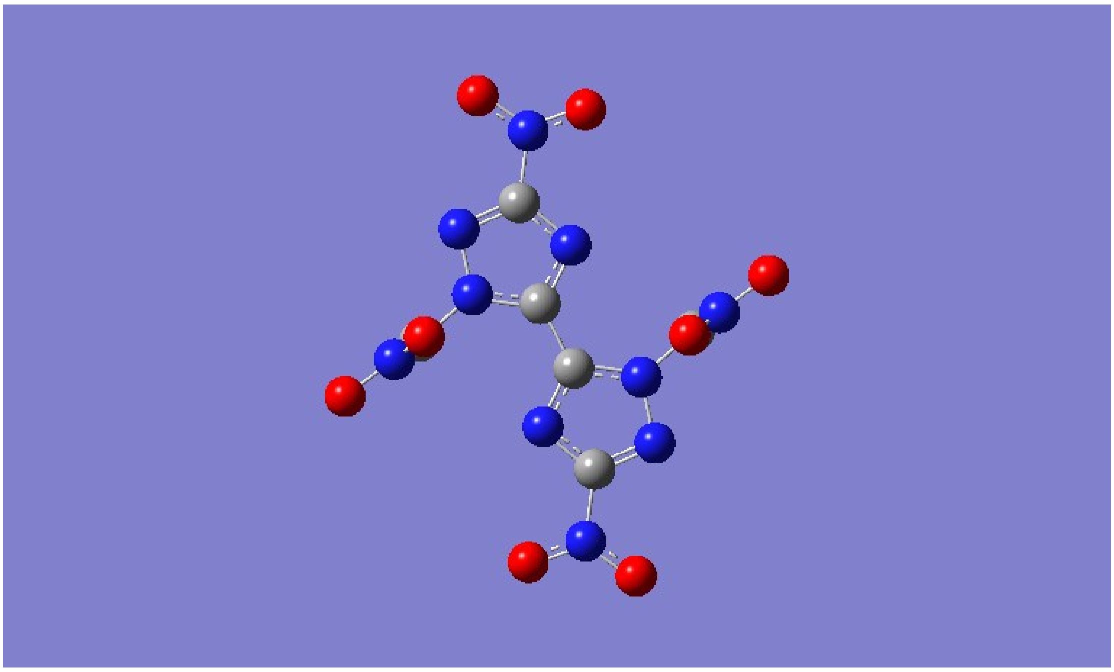

Anion D2

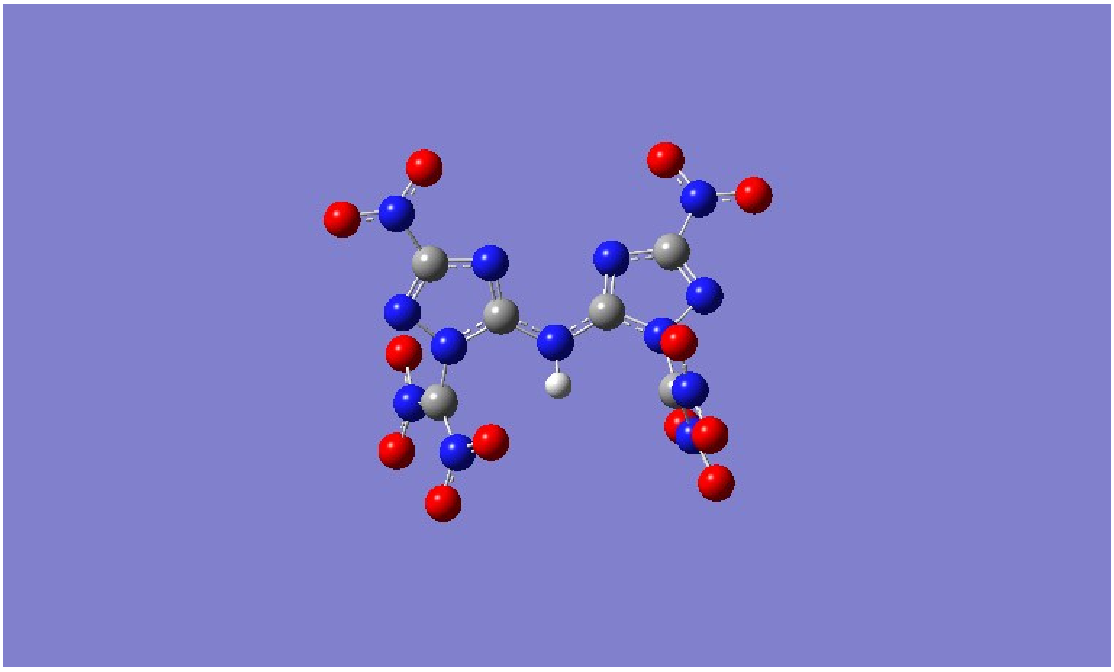

Anion D3

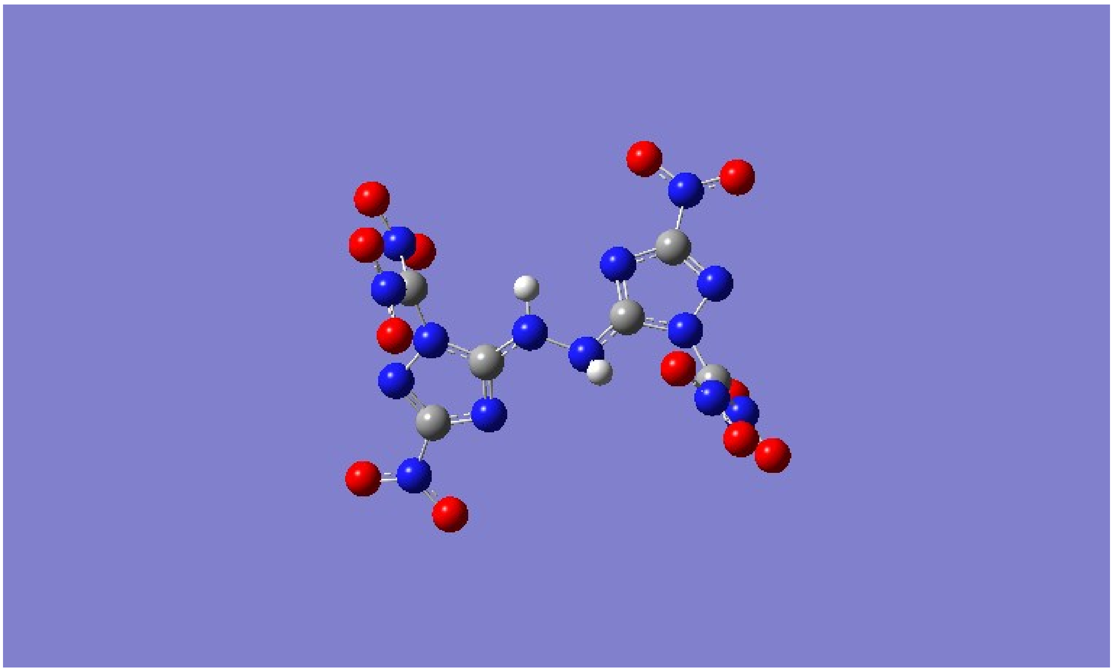

Anion D4

Anion D5

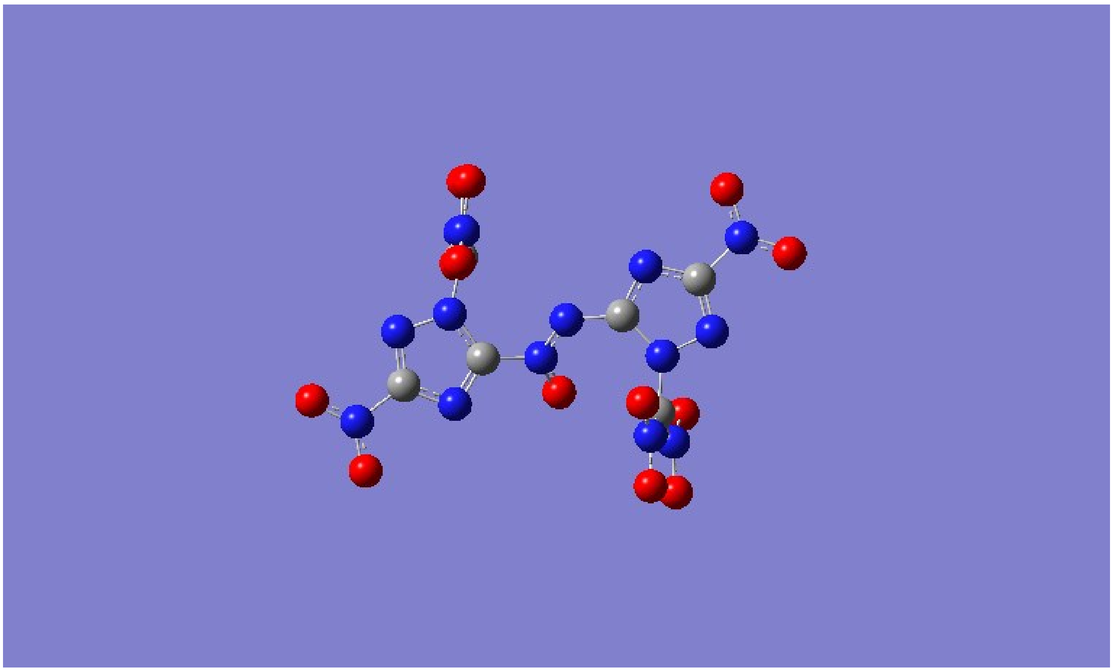

Anion D6

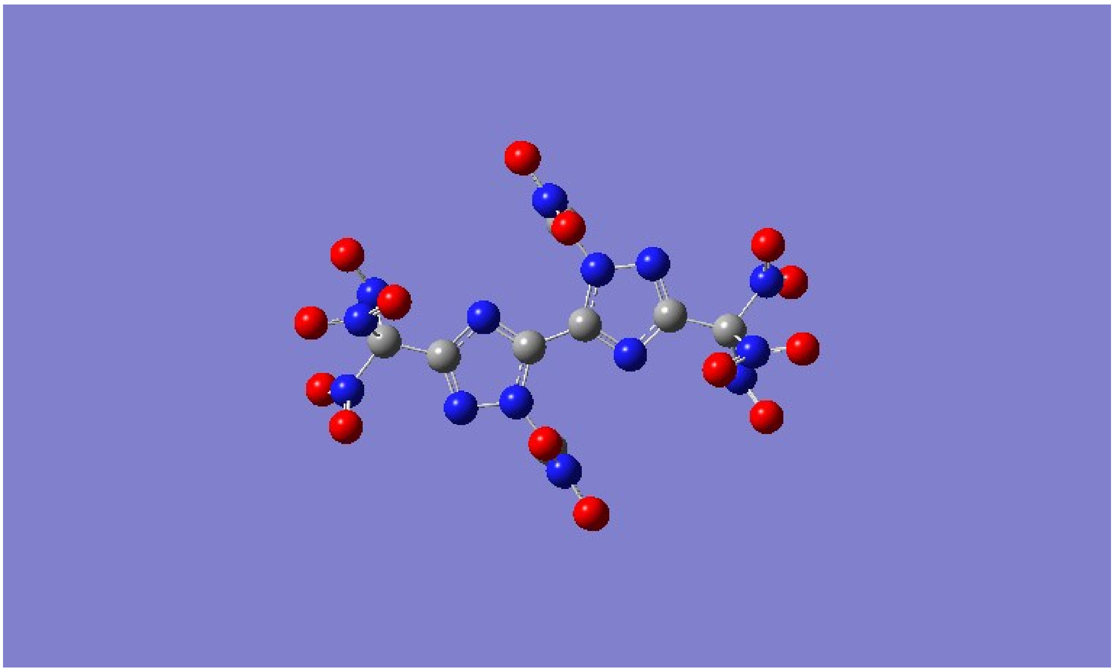

Anion D7

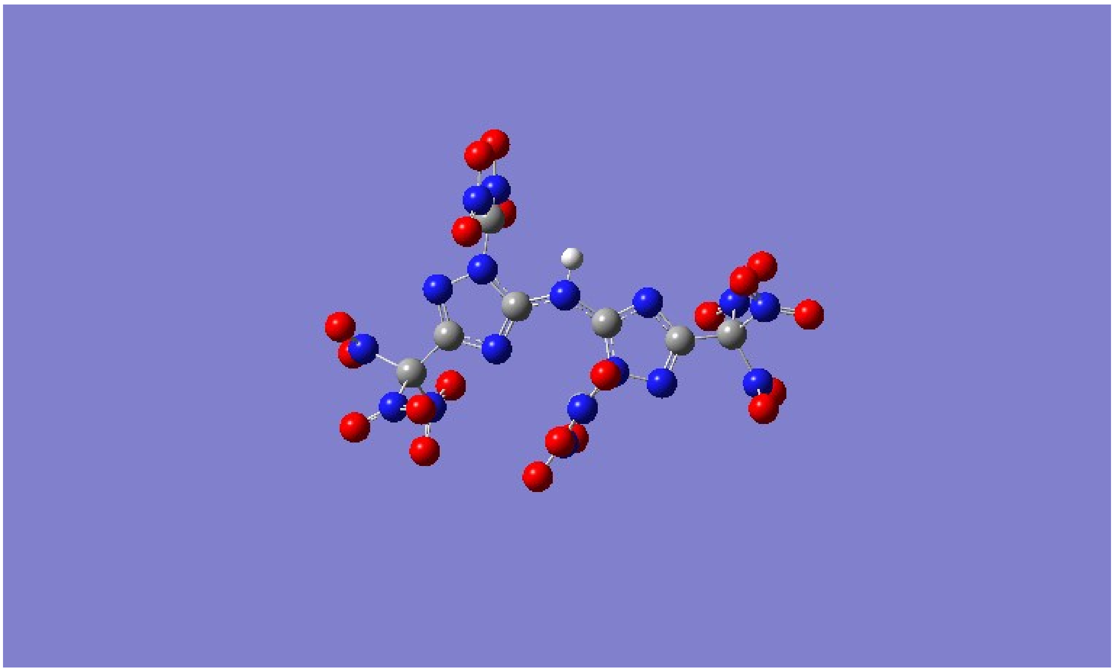

Anion D8

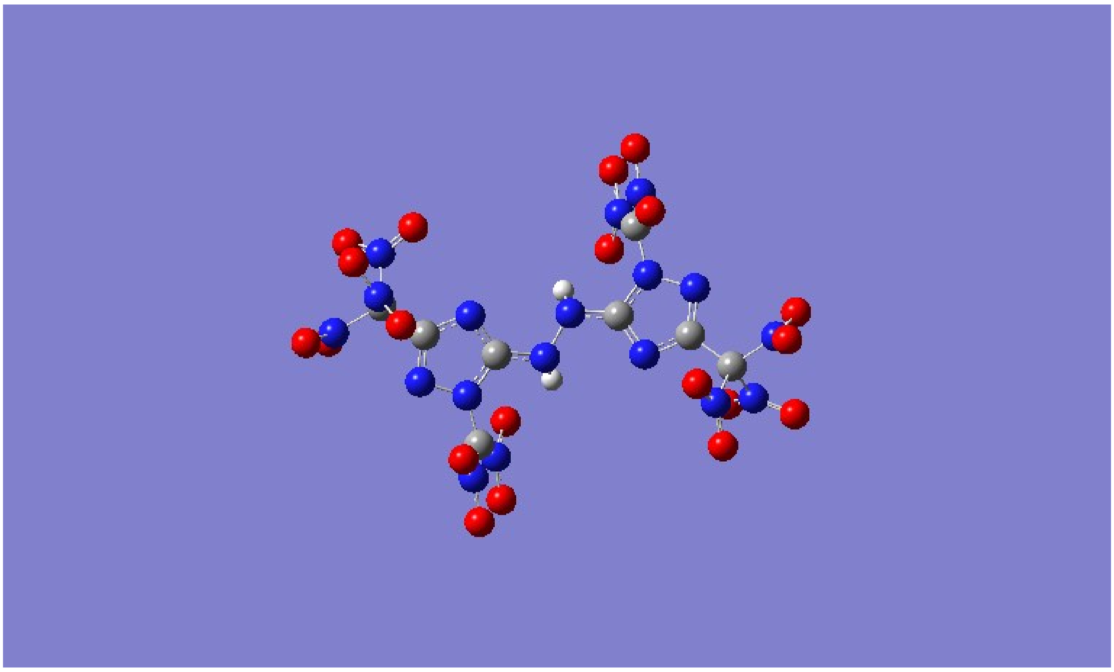

Anion D9

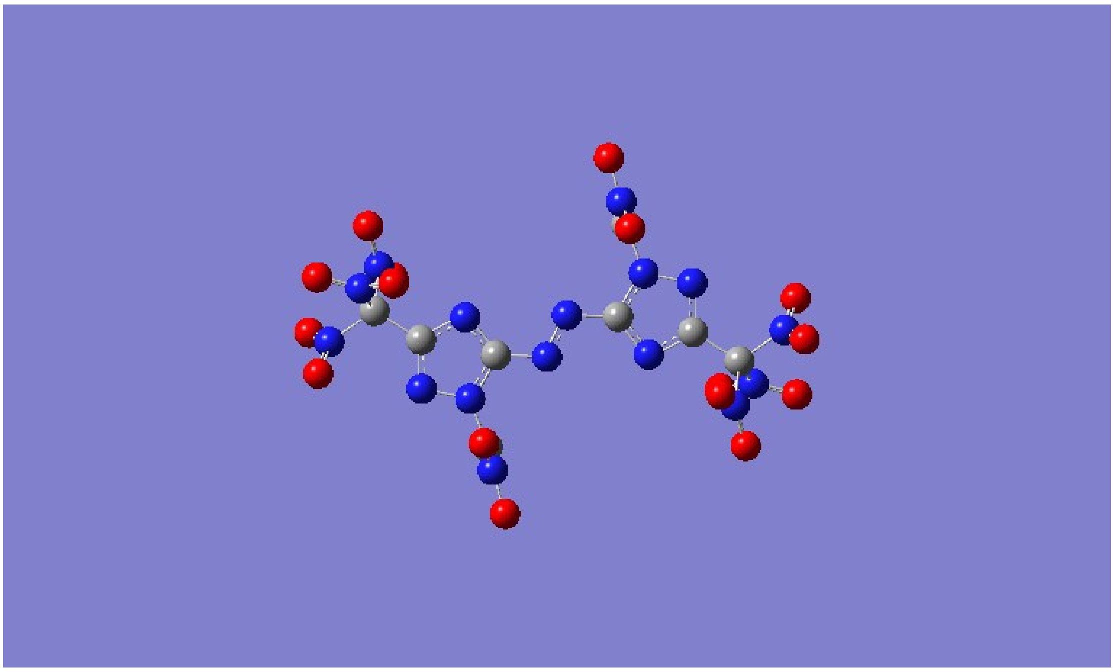

Anion D10

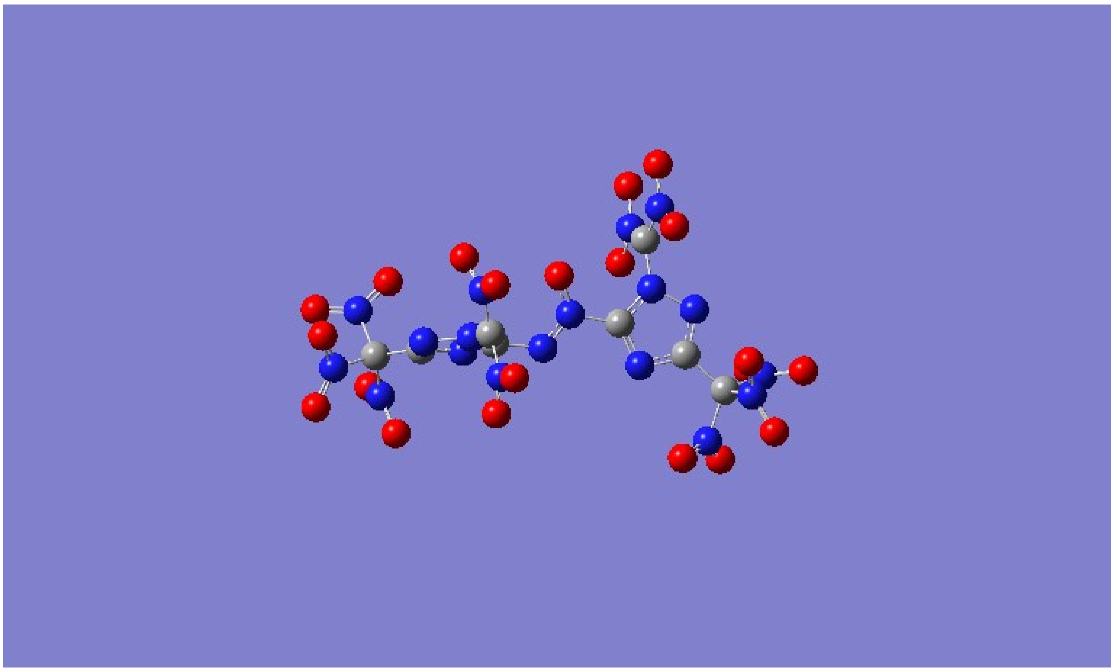

Cation

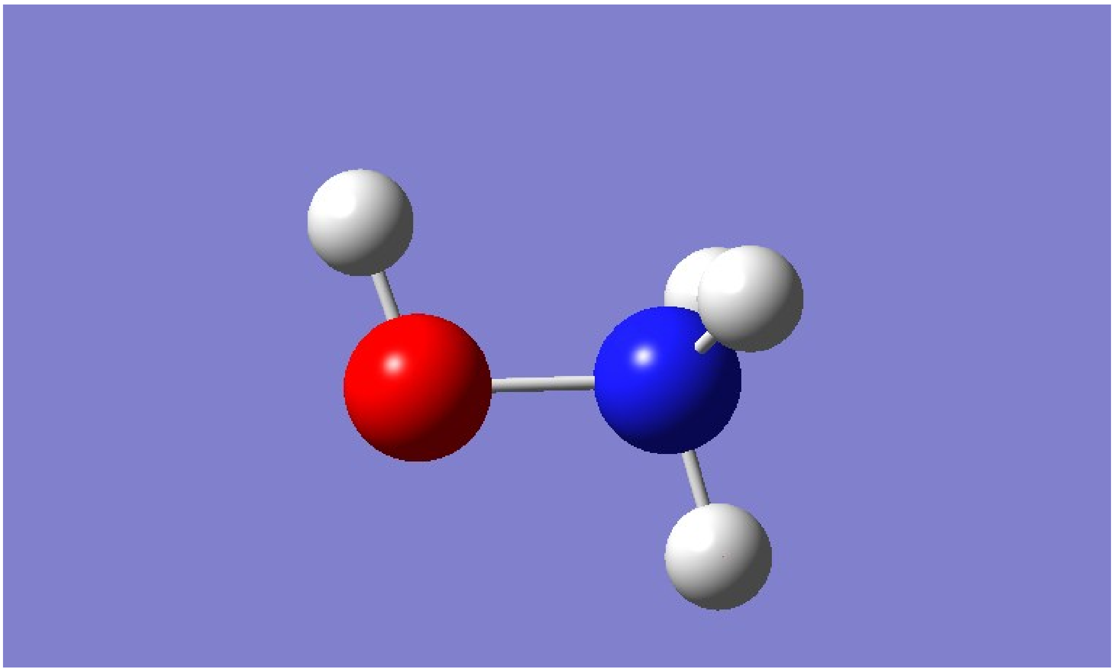

Figure S1. Optimized structures of the related anions and cation

References

1. Ma, Q.; Gu, H.; Huang, J.; Nie, F.; Fan, G.; Liao, L.; Yang, W., Formation of trinitromethyl functionalized 1,2,4-triazole-based energetic ionic salts and a zwitterionic salt directed by an intermolecular and intramolecular metathesis strategy. *New Journal of Chemistry* **2018,** *42* (4), 2376-2380.

2. Dippold, A. A.; Klapotke, T. M., A study of dinitro-bis-1,2,4-triazole-1,1'-diol and derivatives: design of high-performance insensitive energetic materials by the introduction of N-oxides. *Journal of the American Chemical Society* **2013,** *135* (26), 9931-8.

3. Huang, S.; Tian, J.; Qi, X.; Wang, K.; Zhang, Q., Synthesis of gem-Dinitromethylated and Fluorodinitromethylated Derivatives of 5,5'-Dinitro-bis-1,2,4-triazole as Promising High-Energy-Density Materials. *Chemistry-A European Journal* **2017,** *23* (52), 12787-12794.

4. Yin, P.; Shreeve, J. M., From N-Nitro to N-Nitroamino: Preparation of High-Performance Energetic Materials by Introducing Nitrogen-Containing Ions. *Angewandte Chemie International Edition* **2016,** *54* (48), 14513-14517.

5. Xiang, F.; Zhu, W.; Xiao, H., Theoretical studies of energetic nitrogen-rich ionic salts composed of substituted 5-nitroiminotetrazolate anions and various cations. *Journal of Molecular Modeling* **2013,** *19* (8), 3103-3118.

6. Yang, G., Amine Salt–Catalyzed Synthesis of 5-Substituted 1-Tetrazoles from Nitriles. *Synthetic*

Communications **2010,** *40* (17), 2624-2632.
